# Multi-omics analysis reveals a macrophage-related marker gene signature for prognostic prediction, immune landscape, genomic heterogeneity, and drug choices in prostate cancer

**DOI:** 10.3389/fimmu.2023.1122670

**Published:** 2023-04-14

**Authors:** Weian Zhu, Jianjie Wu, Jiongduan Huang, Dongming Xiao, Fengao Li, Chenglun Wu, Xiaojuan Li, Hengda Zeng, Jiayu Zheng, Wenjie Lai, Xingqiao Wen

**Affiliations:** ^1^ Department of Urology, The Third Affiliated Hospital, Sun Yat-sen University, Guangzhou, China; ^2^ Department of Urology, Anqing First People’s Hospital of Anhui Medical University, Anqing, China; ^3^ Department of Health Care, Shenzhen Hospital, Southern Medical University, Shenzhen, China; ^4^ Laboratory of Biomaterials and Translational Medicine, Center for Nanomedicine, The Third Affiliated Hospital, Sun Yat-sen University, Guangzhou, China

**Keywords:** prostate cancer, macrophage-related marker gene, prognostic signature, tumor immunity, genomic heterogeneity, drug choices, single-cell RNA-sequencing

## Abstract

**Introduction:**

Macrophages are components of the innate immune system and can play an anti-tumor or pro-tumor role in the tumor microenvironment owing to their high heterogeneity and plasticity. Meanwhile, prostate cancer (PCa) is an immune-sensitive tumor, making it essential to investigate the value of macrophage-associated networks in its prognosis and treatment.

**Methods:**

Macrophage-related marker genes (MRMGs) were identified through the comprehensive analysis of single-cell sequencing data from GSE141445 and the impact of macrophages on PCa was evaluated using consensus clustering of MRMGs in the TCGA database. Subsequently, a macrophage-related marker gene prognostic signature (MRMGPS) was constructed by LASSO-Cox regression analysis and grouped based on the median risk score. The predictive ability of MRMGPS was verified by experiments, survival analysis, and nomogram in the TCGA cohort and GEO-Merged cohort. Additionally, immune landscape, genomic heterogeneity, tumor stemness, drug sensitivity, and molecular docking were conducted to explore the relationship between MRMGPS and the tumor immune microenvironment, therapeutic response, and drug selection.

**Results:**

We identified 307 MRMGs and verified that macrophages had a strong influence on the development and progression of PCa. Furthermore, we showed that the MRMGPS constructed with 9 genes and the predictive nomogram had excellent predictive ability in both the TCGA and GEO-Merged cohorts. More importantly, we also found the close relationship between MRMGPS and the tumor immune microenvironment, therapeutic response, and drug selection by multi-omics analysis.

**Discussion:**

Our study reveals the application value of MRMGPS in predicting the prognosis of PCa patients. It also provides a novel perspective and theoretical basis for immune research and drug choices for PCa.

## 1 Introduction

Prostate cancer (PCa) is one of the most common genitourinary malignant tumors in the United States, accounting for nearly a quarter of all new diagnoses of male cancers (1). Although continuous improvements have been made in the diagnosis and treatment of PCa in the past decade, the incidence of PCa is still increasing for a variety of reasons. In addition, most patients with advanced PCa will eventually experience biochemical recurrence (BCR) or even death because of their resistance to androgen deprivation therapy and chemotherapy (2, 3). Previous studies have demonstrated that PCa is an immune-sensitive tumor, and immunotherapy is feasible for PCa (4, 5). However, due to the lack of suitable tumor immune biomarkers, most PCa patients have not benefited from immunotherapy at present (6). Therefore, finding appropriate biomarkers to predict prognosis and immunotherapeutic response in PCa patients is of great significance.

As we know, the tumor microenvironment (TME) is a heterogeneous and complex ecosystem that is closely related to the malignant biological process and drug resistance of tumors (7–9). Further understanding of TME is helpful to predict the prognosis of tumors for its feasibility as a biomarker (10). Macrophages are not only powerful immune effector cells in the normal human microenvironment, but also the immune cell components of TME (11). Because of their high heterogeneity and plasticity, macrophages can show anti-tumor or pro-tumor functions in different environments (12, 13). At the same time, tumor-associated macrophages (TAMs) are considered to be pro-tumoral macrophages, which can inhibit T cell-mediated anti-tumor immune response, and then promote the initiation and metastasis of tumor cells (14). Recent studies have found that TAMs are positively correlated with tumorigenesis and shorter biochemical recurrence-free survival (bRFS) in PCa patients (15–17). Based on the vital role of macrophages in tumor behavior, it is necessary to accurately establish a more specific predictive signature for macrophages to better prognostic prediction and therapeutic response of patients with PCa.

Single-cell RNA-sequencing (scRNA-seq) is a novel technique to amplify and sequence the whole genome at the single-cell level, which can be used to explore the abundance and functional status of cell types and analyze the molecular characteristics of macrophages in TME (18, 19). Furthermore, compared with traditional transcriptome analysis, it can not only evaluate thousands of cells in one sample at the same time but also fully reveal the heterogeneity between tumor cells and the complexity of TME (20). In recent years, this technique has been widely used to explore the clinical treatment and tumor characteristics of PCa (21, 22). Thus, with these huge advantages, we can construct the prognostic signature of PCa according to the specific cell type such as macrophages, and provide a novel treatment strategy.

In our study, we aimed to identify macrophage-related marker genes (MRMGs) and explore their significance in the occurrence and development of PCa based on the scRNA-seq and unsupervised clustering algorithm. Subsequently, we further constructed and verified a macrophage-related marker gene prognostic signature (MRMGPS) for assessing the prognosis of PCa. In addition, we also revealed the relationship between MRMGPS and tumor immune microenvironment, genomic heterogeneity, therapeutic response, and the value of drug choices in PCa. In conclusion, these findings may provide reliable biomarkers and therapeutic strategies for the clinical treatment of PCa.

## 2 Materials and methods

### 2.1 Data sources and preprocessing

The 13 PCa samples with scRNA-seq data (GSE141445) were downloaded from the Gene Expression Omnibus (GEO, https://www.ncbi.nlm.nih/) and were used to identify MRMGs. In addition, a total of 548 samples were downloaded from The Cancer Genome Atlas (TCGA, https://portal.gdc.cancer.gov/) including 496 PCa samples and 52 normal samples. After eliminating incomplete data, 346 PCa samples were included in the analysis as a training cohort. Subsequently, to evaluate the predictive accuracy of the model in a verification cohort, GSE70768 (n=110) and GSE46602 (n=36) collected from the GEO database were merged and eliminated batch effects by R package “inSilicoMerging” and empirical Bayes methods (23).

### 2.2 Identification of MRMGs by scRNA-seq analysis

We used the “Seurat” package in R to generate objects and filter out high-quality cells. The filtering criteria were to remove the genes from less than 3 cells, the cells with less than 50 genes detected, or the cells with more than 5% mitochondria genes. The data were then normalized using the “LogNormalize” method, and the first 1500 highly variable genes identified by JackStraw analysis were analyzed by principal component analysis (PCA). Furthermore, the “FindClusters” function (resolution=0.5) was used to cluster and visualize the first 15 PCA in t-distributed stochastic neighbor embedding (t-SNE). Finally, the marker genes of each cluster were identified (cut-off thresholds: adjusted *P*-value < 0.05 and |logFC| > 1) by comparing the differences of gene expression between a cluster and all other clusters using the “FindAllMarkers” function and Wilcoxon-Mann-Whitney test, and the cell types were annotated and visualized by the “SingleR” package.

### 2.3 Consensus clustering of MRMGs

Based on the MRMGs, we utilized the “ConensusClusterPlus” package in R for consensus molecular clustering methods and divided the patients from the TCGA cohort into different clusters. After 1000 initial resamples and 50 iterations by the unsupervised clustering algorithm, the most optimal clustering number was selected according to the consistency matrix, the cumulative distribution function (CDF), and the relative change of the area under the CDF curve.

Subsequently, the differences and bRFS rates among different clusters were computed and visualized by the R packages of “scatterplot3d”, “survival”, and “survminer”. The relationships between different clusters and the clinicopathological characteristics (age, Gleason score, prostate specific antigen (PSA), pathological T stage, and pathological N stage) were analyzed with the chi-square test. Besides, the differences of somatic mutations from TCGA in different clusters were analyzed using the R package “maftools”, and a single-sample GSEA (ssGSEA) algorithm with the “GSVA” package in R was utilized to estimate the infiltration of immune cells in each sample based on the gene sets of immune cell types obtained from the previous study (24).

### 2.4 Functional analysis

Based on the differentially expressed genes (DEGs) identified *via* the “LIMMA” package in R (FDR < 0.05) among three MRMGs clusters or between high- and low-risk groups, the “clusterProfiler” R package was used to perform gene ontology (GO) and Kyoto Encyclopedia of Genes and Genomes (KEGG) analysis (25), and *P*-value < 0.05 was considered to be significant enrichment. *P*-value was adjusted with the Benjamini-Hochberg methods.

Gene set enrichment analysis (GSEA) was used to analyze the relative richness of specific gene sets in the sample population, and to detect statistical differential expression patterns among three MRMGs clusters or between high- and low-risk groups, with FDR <0.05.

### 2.5 Construction and validation of prognostic signature

Based on the MRMGs identified by scRNA-seq analysis, the prognostic MRMGs were identified as candidate genes by univariate Cox regression analysis. The DEGs between PCa tumors and normal samples from the TCGA were selected through Wilcoxon test by the R package of “LIMMA” (FDR < 0.05). Then the intersection of scRNA-MRMGs, prognostic MRMGs, and DEGs was obtained using the “Venn” package in R. Meanwhile, a protein-protein interaction (PPI) network of the intersection was built by the STRING database (https://string-db.org/) (26) and visualized by Cytoscape (version 3.8.2) (27). The intersection genes were evaluated by least absolute shrinkage and selection operator (LASSO) Cox proportional hazards regression using the “glmnet” package to eliminate the effect of overfitting. Then, the criterion for identifying optimal model genes: According to the minimum Akaike information criterion (AIC), the optimal model genes were obtained by taking the optimal penalty parameter (λ) corresponding to the minimum 1-standard error (SE) obtained from the 10-fold cross-validation results (28).

Subsequently, the risk score was calculated by summing the expression and coefficient of each gene of MRMGPS. The distribution of signature genes within different cell types was further visualized by the R packages of “Vlnplot”, “Dimplot” and “Featureplot” at the single-cell level, and the CIBERSORT algorithm was used to estimate the relative proportion of macrophage proportions deconvoluted from the TCGA cohort and then calculate the correlation with gene expression. According to the median value of risk score, patients were divided into two groups with low- or high-risk score. Furthermore, t-SNE and PCA were performed to reveal distribution between two groups by the packages of “stats” and “Rtsne”. Receiver operating characteristic (ROC) curves and Kaplan-Meier curves were generated to evaluate the accuracy of MRMGPS by “timeROC”, “surviminer” and “survival” in R. To further verify the accuracy and reliability of the novel signature, the merged dataset (GSE70768 and GSE46602), named GEO-Merged cohort, was conducted to verify the reliability of the signature in the same way as the TCGA cohort.

### 2.6 Real-time quantitative PCR and immunohistochemistry analysis

20 fresh PCa and 20 benign prostatic hyperplasia (BPH) tissue samples were collected from the Third Affiliated Hospital of Sun Yat-sen University (Guangzhou, China) from 2021 to 2022. All the selected samples were informed consent of the patients and approved by the Clinical Ethics Board of the Third Affiliated Hospital of Sun Yat-sen University. Following a standard protocol, total RNA was extracted by TRIzol reagent (Invitrogen, Cat #15596018) and reverse transcribed by SuperScript™ III Reverse Transcriptase (Invitrogen, Cat # 18080044). SYBR Green qPCR reagent (GenStar, Cat #A311) was applied for RT-qPCR. *GAPDH* was used as the internal control. The primers used for RT-qPCR were listed in **Table S1**.

The IHC data and images collected from the Human Protein Atlas (HPA, https://www.proteinatlas.org/) (29) were used to reveal expression levels of protein of signature genes between PCa and normal samples.

### 2.7 Construction and validation of nomogram

The differences between MRMGPS-based risk score and clinicopathological features were analyzed by a chi-square test, and stratified survival analysis of patients in two subgroups was conducted to evaluate the robustness of MRMGPS in both TCGA and GEO-Merged cohorts.

Meanwhile, independent prognostic indicators were determined by COX proportional hazard regression model and the independent indicators were used to construct nomogram for predicting 1-, 3-, and 5-year bRFS by using the “rms” package. Furthermore, decision curve analysis (DCA), ROC curves, and calibration curves were performed to assess the accuracy and discrimination of the nomogram.

### 2.8 Immune landscape analysis

Firstly, the immune infiltration and function of two groups based on MRMGPS were calculated by the EPIC (30), XCell (31), MCPCOUNTER (32), QUANTISEQ (33), CIBERSORT-ABS, CIBERSORT (34), and TIMER (35) algorithms. Secondly, the ssGSEA algorithm with the “GSVA” package and Mann-Whitney test were utilized to calculate the infiltration scores of 29 immune cells or pathways obtained from the previous study (36). The gene sets with annotation were shown in **Table S2**. Thirdly, the ESTIMATE scores, immune scores, stromal scores, and tumor purity of the two groups were estimated and compared by the R package of “estimate” (37). Finally, the scores of 7 steps in the cancer-immunity cycle based on MRMGPS were analyzed by Tracking Tumor Immunophenotype (TIP, https://biocc.hrbmu.edu.cn/TIP/) as in previous studies (38).

### 2.9 Genomic heterogeneity and tumor stemness analysis

The Copy Number Variation (CNV) data of all PCa samples in TCGA processed by GISTIC software (39) were downloaded from GDC (https://portal.gdc.cancer.gov/), and further integrated for analysis and visualization. The somatic mutation data in TCGA processed by the VarScan platform were analyzed between two groups by the package of “maftools” and the tumor mutational burden (TMB) between the two groups was compared. In addition, the markers of genomic heterogeneities, including homologous recombination defect (HRD), microsatellite instability (MSI), mutant-allele tumor heterogeneity (MATH), loss of heterozygosity (LOH), ploidy, neoantigens, and RNA-modified genes (**Table S3**), were collected from previous studies (40). The markers of tumor stemness, including DNA-methylation-based stemness scores (DNAss) and RNA-based stemness scores (RNAss), were collected from the study of Malta TM (41). Furthermore, the correlation between them and MRMGPS-based risk score were analyzed by Pearson or Mann–Whitney methods.

### 2.10 Therapeutic responses analysis

The differences in risk score and 46 immune checkpoints obtained from the previous research (42) were examined to explore the relationship. In addition, the immunophenoscore (IPS) of PCa samples in TCGA were collected from The Cancer Immunome Atlas (https://tcia.at/) (24) and were used to compare the responses of *PD1* and *CTLA4* in two subgroups.

### 2.11 Drug sensitivity and molecular docking

According to the IC50 value from the Genomics of Drug Sensitivity in Cancer (https://www.cancerrxgene.org/) (43), the efficacy of common anticancer drugs between two subgroups was calculated by the “pRRophetic” in R. Besides, the pharmacological information about the anticancer drug targets was collected from DrugBank database (https://go.drugbank.com/) (44), and was used to reveal their expression level in different groups.

The molecular structures of the targeted drugs were retrieved from PubChem (https://pubchem.ncbi.nlm.nih.gov/) (45) and the coordinates of MRMGPS-based genes were obtained from Protein Data Bank (PDB, http://www.rcsb.org/pdb/home/home.do) (46). A Molecular Operating Environment (MOE, version 2019.0102) was used to exclude all water molecules from the protein and ligand files and then add polar hydrogen atoms. Finally, molecular computing and docking were performed to assess the binding energy and interaction mode between targeted drugs and MRMGPS-based genes.

### 2.12 Statistical analysis

This study used R software (version 4.2.1) for data analysis and graphic visualization. For quantitative data, an independent *t*-test was utilized to analyze normal distribution variables, and the Wilcoxon test was used to analyze non-normal distribution variables. For qualitative data, a one-way analysis of variance was used to analyze ordinal categories variables, and the Kruskal-Wallis test was utilized to analyze unordered categorical variables. Kaplan-Meier survival analysis was performed by the log-rank test. For all tests, the value of *P* less than 0.05 was considered statistically significant (*P <0.05, **P < 0.01, ***P < 0.001, ****P < 0.0001. ns, not significant).

## 3 Results

**Figure 1:**
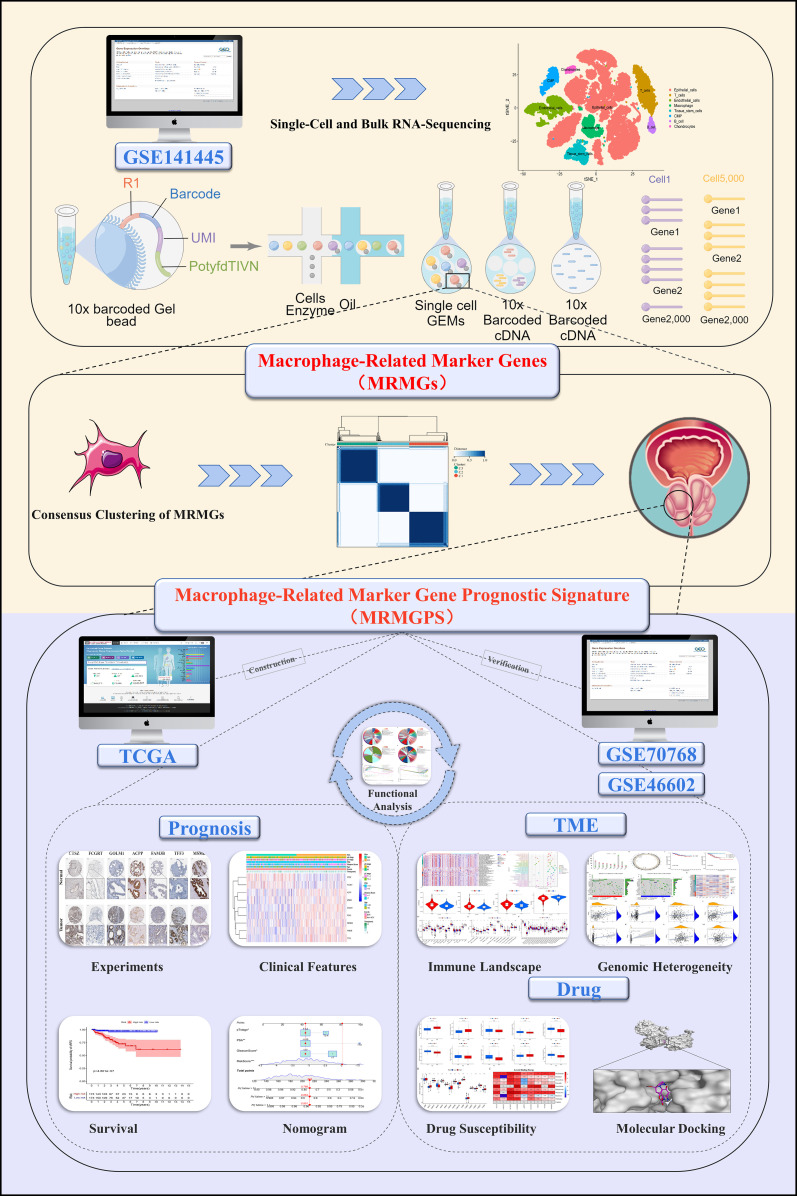
The flow chart of the present study. Mining MRMGs through scRNA-seq data to construct a MRMGPS to predict prognosis, analyze TME, and select drugs in PCa. TCGA, The Cancer Genome Atlas; scRNA-seq, single-cell RNA-sequencing; MRMGs, macrophage-related marker genes; MRMGPS, macrophage-related marker gene prognostic signature; TME, tumor microenvironment; PCa, prostate cancer.

The flow chart of the research was shown in **Figure 1**. Meanwhile, the main clinicopathological features of patients with PCa included in the present study were listed in **Table S4**.

### 3.1 Definition of MRMGs by scRNA-seq

**Figure 2:**
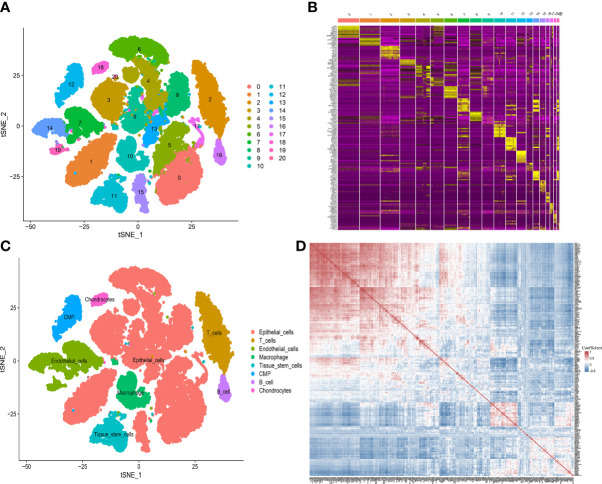
Definition of MRMGs by scRNA-seq analysis. **(A)** t-SNE plot displaying 21 cell-related clusters. **(B)** Heatmap displaying top 10 marker genes in 21 clusters (https://postimg.cc/2bK9wfXT). **(C)** t-SNE plot displaying 8 annotated cell types. **(D)** Heatmap displaying the correlation between 307 identified MRMGs (https://postimg.cc/cvFkkdVY). t-SNE, t-distributed stochastic neighbor embedding; CMP, common myeloid progenitors.

By analyzing the scRNA-seq data from GSE141445, we collected gene expression profiles of 33441 single cells from 13 PCa samples. Then 21 cell clusters were identified by PCA analysis, and the cells in cluster 10 were defined as macrophage (**Table S5**). Moreover, a total of 307 genes were differentially expressed in the macrophage cluster, which were defined as MRMGs and used for further research (**Figures 2A–C**). The correlation of 307 MRMGs was presented in the heatmap (**Figure 2D**).

### 3.2 Consensus clustering and phenotypic analysis of MRMGs in PCa

**Figure 3:**
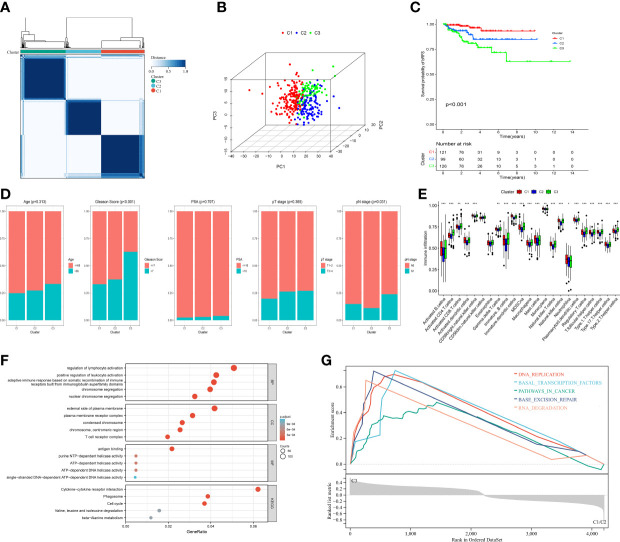
Identification of MRMGs clusters and phenotypic differences in PCa. **(A)** The consensus clustering of 307 MRMGs in three clusters (C1 = 121, C2 = 99, C3 = 126). **(B)** 3D-PCA plot displaying the distribution among three clusters. **(C)** Kaplan–Meier curves for the probability of bRFS grouped in three clusters. **(D)** Comparison of the clinicopathological features among three clusters. **(E)** Comparison of the immune infiltration among three clusters. **(F)** GO and KEGG analysis based on MRMGs-related DEGs among three clusters. **(G)** GSEA analysis based on MRMGs-related DEGs among three clusters. PCA, principal component analysis; bRFS, biochemical recurrence-free survival; GO, gene ontology (BP, biological processes; CC, cellular components; MF, molecular functions); KEGG, kyoto encyclopedia of genes and genomes; DEGs, differentially expressed genes; GSEA, gene set enrichment analysis. *P <0.05, **P < 0.01, ***P < 0.001.

To explore the expression features of MRMGs, we first performed PCA analysis based on the expression of MRMGs between PCa and normal samples in TCGA. The results suggested that individuals could be distinguished from PCa to normal (**Supplementary Figure 1A**), indicating different regulatory roles of macrophages in normal and PCa. Based on the expression of MRMGs, we clustered the consensus of the patients in the TCGA cohort, and according to the area under the line of the CDF curve, the downward trend of CDF delta, and average consistency within the cluster groups (**Supplementary Figures 1B–D**), we obtained optimal three clusters (k=3, C1 = 121, C2 = 99, C3 = 126) (**Figure 3A**). Further PCA analysis showed a distinct clustering, indicating a notable differential distribution of the MRMGs in three clusters (**Figure 3B**). Survival analysis revealed that the patients in C3 suffered the worst prognosis, whereas the patients in C1 had the best prognosis (*P* < 0.001) (**Figure 3C**). In addition, the analysis of the relationship among different clusters and clinical features showed that C3 had a higher Gleason score (*P* < 0.001) and an advanced pathological N stage (*P* = 0.031) (**Figure 3D**), which explained the poor prognosis.

Meanwhile, different clusters also showed remarkable diversity in somatic mutations and immune phenotypes (**Supplementary Figure 1E**). The level of immune infiltration in 13 out of 23 immune cells in C3 was significantly higher than that in C1 and C2 (**Figure 3E**). Hence, we hypothesized that macrophages played a significant role in the features of immunological infiltration based on the aforementioned data.

Subsequently, to explore the differences in biological function among different clusters, we carried out an enrichment analysis based on 4200 DEGs among three MRMGs clusters. These DEGs were not only enriched in biochemical processes related to the regulation of lymphocyte activation and cytokine receptor interaction pathways but also GSEA showed that C3 was likely to be involved in cancer-related pathways explaining the worse prognosis of C3 (**Figures 3F, G** **)**. In general, our data suggested that macrophages played a significant role in the progression of PCa, and we had enough reason to predict that MRMGs might provide important prognostic information.

### 3.3 Construction of MRMGPS in TCGA cohort

To select the genes with prognostic value from scRNA-MRMGs for further studying, we first selected 21 overlapping genes among 307 scRNA-MRMGs, 18304 DEGs between PCa and normal samples, and 38 prognostic MRMGs (**Supplementary Figures 2A–D**). Then, through LASSO-Cox regression analysis based on the minimum AIC (λ = 0.03), we finally constructed an optimal signature containing nine genes (*CTSZ*, *FCGRT*, *GOLM1*, *SMIM22*, *ACPP*, *FAM3B*, *TFF3*, *PCA3*, and *MSMB*), named MRMGPS (**Supplementary Figures 2E, F** **)**. Based on their coefficients, MRMGPS-based risk score was built: Risk score = (0.411 × *CTSZ*) + (0.025 × *FCGRT*) + (-0.161 × *GOLM1*) + (-0.107 × *SMIM22*) + (-0.012 × *ACPP*) + (-0.029 × *FAM3B*) + (-0.056 × *TFF3*) + (-0.025 × *PCA3*) + (-0.080 × *MSMB*).

According to the formula for calculating the risk score, it could be considered that the risk score is largely determined by the expression of *CTSZ*, which was mainly expressed in immune cells such as macrophages and monocytes (47). Therefore, we analyzed the expression profiles of model genes across all cell types identified in the scRNA-seq data and further calculated the correlation between *CTSZ* expression and macrophage proportions deconvoluted from the bulk RNA-seq data of TCGA cohort. The results showed that *CTSZ* was mainly expressed in macrophages (**Supplementary Figures 3A–C**) and positively correlated with macrophage proportions (R = 0.11, *P* = 0.04) (**Supplementary Figure 3D**), suggesting the reliability of our formula for calculating the risk score.

**Figure 4:**
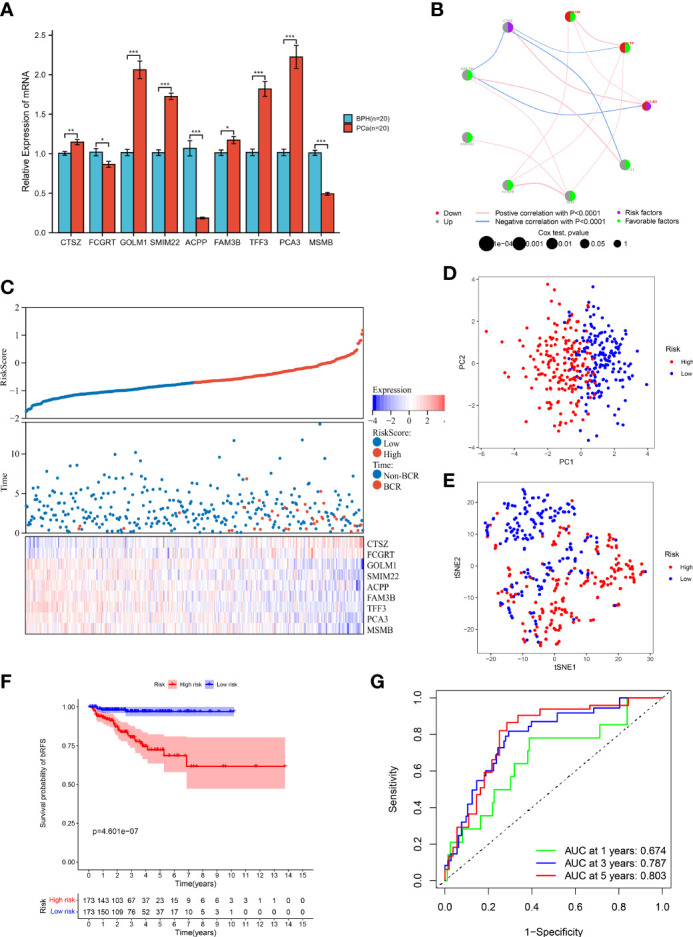
Prognostic value of MRMGPS in TCGA cohort. **(A)** The mRNA levels of MRMGPS-based genes in 20 fresh PCa and 20 BPH tissue samples detected by RT-qPCR. **(B)** Interaction between signature genes in PCa. The lines connecting the genes represent their interactions with the thickness representing the strength of the association and the color representing blue-negative or pink-positive associations. The color of the left dots represents the grey-upregulation or red-downregulation of the genes and the color of the right dots represents the purple-risk factors or green-favorable factors of the genes. The size of dots represents the effect of each gene on the prognosis. **(C)** Relationship between risk score and follow-up time, BCR events, and changes of model genes expression in PCa patients. **(D)** PCA plot displaying the distribution of high- and low-risk groups. **(E)** t-SNE plot displaying the distribution of high- and low-risk groups. **(F)** Kaplan–Meier curves for the probability of bRFS grouped in high- and low-risk groups. **(G)** ROC curves for the predictive efficiency in 1-, 3-, and 5-year bRFS of MRMGPS. BPH, benign prostatic hyperplasia; ROC, receiver operating characteristic; AUC, area under the ROC curve; BCR, biochemical recurrence. *P <0.05, **P < 0.01, ***P < 0.001.

We further examined the mRNA expression profiles of the model genes by RT-qPCR. The result demonstrated that *CTSZ*, *GOLM1*, *SMIM22*, *FAM3B*, *TFF3*, and *PCA3* had higher expression, while *FCGRT*, *ACPP*, and *MSMB* had lower expression in PCa than BPH samples (**Figure 4A**). We also observed consistent trends in the protein levels of *CTSZ*, *FCGRT*, *GOLM1*, *ACPP*, *FAM3B*, *TFF3*, and *MSMB* by exploring the HPA database (**Supplementary Figure 4**). Unfortunately, there were no matching IHC images of *PCA3* and *SMIM22* in the database. In addition, the network diagram provided a comprehensive view of survival and interactions between model genes (**Figure 4B**).

According to the median risk score, patients were divided into high- or low-risk groups. The risk score plot and survival status indicated that the low-risk group had lower BCR rates (**Figure 4C**). The results of the PCA and t-SNE analysis implied that the distribution of samples in two groups showed two trends (**Figures 4D, E**). Moreover, the Kaplan-Meier curve revealed that patients in the low-risk group had better bRFS (*P* < 0.001) (**Figure 4F**). Consistently, the area under the ROC curve (AUC) for 1-, 3-, and 5-year bRFS were 0.674, 0.787, and 0.803, respectively (**Figure 4G**). The above results showed that MRMGPS had good accuracy in predicting the prognosis of PCa patients.

### 3.4 Validation of MRMGPS in GEO-Merged Cohort

To better verify the robustness of MRMGPS, we combined two independent cohorts (GSE70768 and GSE46602) into an external cohort (GEO-Merged) for evaluation (**Supplementary Figure 5A**). After removing the batch effect, the data distribution tended to be consistent between each dataset. The median was homogenized, the mean and variance were comparable, and the samples between the datasets were clustered and intertwined with each other (**Supplementary Figure 5B**), suggesting that the batch effect was better removed.

The same formula as in the TCGA cohort was used to calculate the risk score in the GEO-Merged cohort. The risk score plot and survival status demonstrated consistent trends as shown in the TCGA cohort (**Supplementary Figure 5C**), and indicated that the risk score was a reliable predictor of BCR in patients with PCa. Consistent with the TCGA cohort, PCA and t-SNE also demonstrated excellent separation between two groups (**Supplementary Figures 5D, E** **)**. Besides, survival analysis demonstrated that individuals in the high-risk group had worse bRFS than patients in the low-risk group (*P* < 0.05) (**Supplementary Figure 5F**). ROC analysis revealed that the AUCs of the MRMGPS were 0.652 at 1 year, 0.685 at 3 years, and 0.700 at 5 years (**Supplementary Figure 5G**).

### 3.5 Relationship between MRMGPS and clinicopathological features

**Figure 5:**
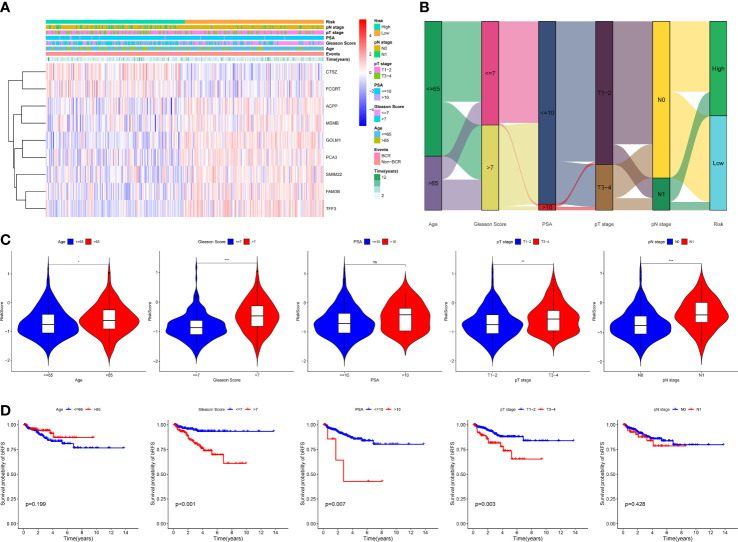
Correlation between MRMGPS and clinicopathological features in TCGA cohort. **(A)** The landscape of MRMGPS and clinicopathological features (pN stage, pT stage, PSA, Gleason Score, Age). **(B)** Sankey diagram displaying the distribution of samples in different subgroups stratified by clinicopathological features. **(C)** Comparison of the risk score between different subgroups stratified by clinicopathological features. **(D)** Kaplan–Meier curves for the probability of bRFS stratified by clinicopathological features. PSA, prostate specific antigen. *P <0.05, **P < 0.01, ***P < 0.001.

To explore the association with clinical and pathological characteristics, we first compared the differences in MRMGPS-based risk score according to different stratified characteristics. The violin charts indicated that patients with advanced age (*P* < 0.05), Gleason score (*P* < 0.001), pathological T stage (*P* < 0.01), and pathological N stage (*P* < 0.001) had higher risk score in TCGA cohort (**Figures 5A–C**). The GEO-Merged cohort showed the same significant differences in Gleason score (*P* < 0.05) and pathological T stage (*P* < 0.05) as the TCGA cohort (**Supplementary Figures 6A–C**). Moreover, we found that both TCGA cohort and GEO-Merged cohort patients with advanced Gleason score (TCGA cohort: *P* < 0.001; GEO-Merged cohort: *P* = 0.003) and pathological T stage (TCGA cohort: *P* = 0.003; GEO-Merged cohort: *P* = 0.008) had poorer bRFS by stratified survival analysis (**Figure 5D**, **Supplementary Figure 6D**).

Next, to explore the independence of MRMGPS-based risk score and clinicopathological factors, we also carried out univariate and multivariate Cox regression analysis. Univariate Cox regression analysis showed that Gleason score, PSA, pathological T stage, and risk score were significantly correlated with bRFS in both TCGA and GEO-Merged cohorts. Multivariate Cox regression analysis showed that they remained independent predictors of bRFS (**Table S6**).

### 3.6 Construction and validation of the prognostic prediction nomogram

**Figure 6:**
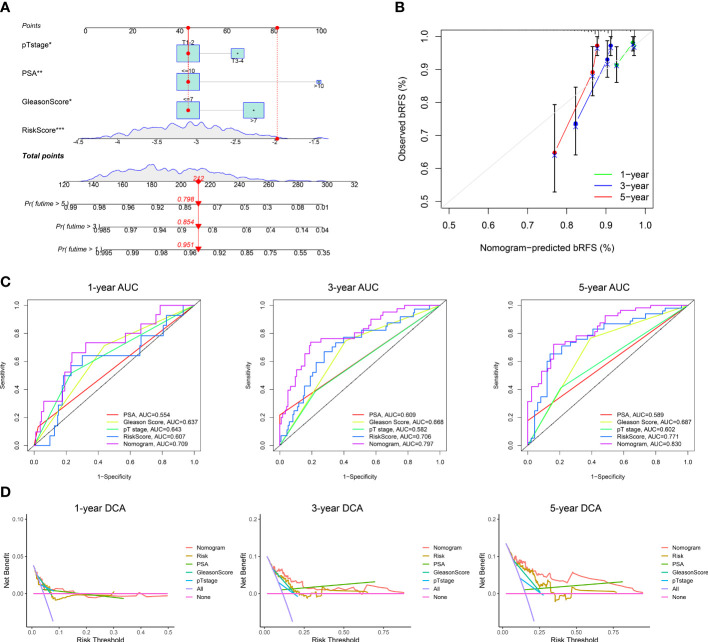
Construction of a nomogram in TCGA cohort. **(A)** The nomogram based on risk score, pT stage, PSA, and Gleason Score. **(B)** Calibration curves for the internal verification in 1-, 3-, and 5-year bRFS of the nomogram. **(C)** ROC curves for the predictive efficiency in 1-, 3-, and 5-year bRFS of the nomogram. **(D)** DCA curves for the net benefit in 1-, 3-, and 5-year bRFS of the nomogram. DCA, decision curve analysis; *P <0.05, **P < 0.01, ***P < 0.001.

To explore the value of MRMGPS in clinical practice, we built a predictive nomogram for bRFS of the independent factors (risk score, pathological T stage, PSA, and Gleason score) obtained by multivariate regression analysis in TCGA and verified by GEO-Merged cohort (**Figure 6A**, **Supplementary Figure 7A**). As expected, the calibration charts of the nomogram showed excellent predictive performance in both the training and validation cohorts (**Figure 6B**, **Supplementary Figure 7B**). The AUCs in both cohorts demonstrated that the nomogram was more effective at predicting bRFS than the risk score, PSA, Gleason score, and pathological T stage alone (TCGA cohort: 1-year = 0.709, 3-year = 0.797, 5-year = 0.830; GEO-Merged cohort: 1-year = 0.656, 3-year = 0.759, 5-year = 0.789) (**Figure 6C**, **Supplementary Figure 7C**). Furthermore, the nomogram had a higher net benefit than other independent factors in two cohorts (**Figure 6D**, **Supplementary Figure 7D**). The findings indicated that the nomogram for bRFS had excellent predictive ability for long-term BCR risk and brought greater application value.

### 3.7 Functional enrichment analysis based on MRMGPS

**Figure 7:**
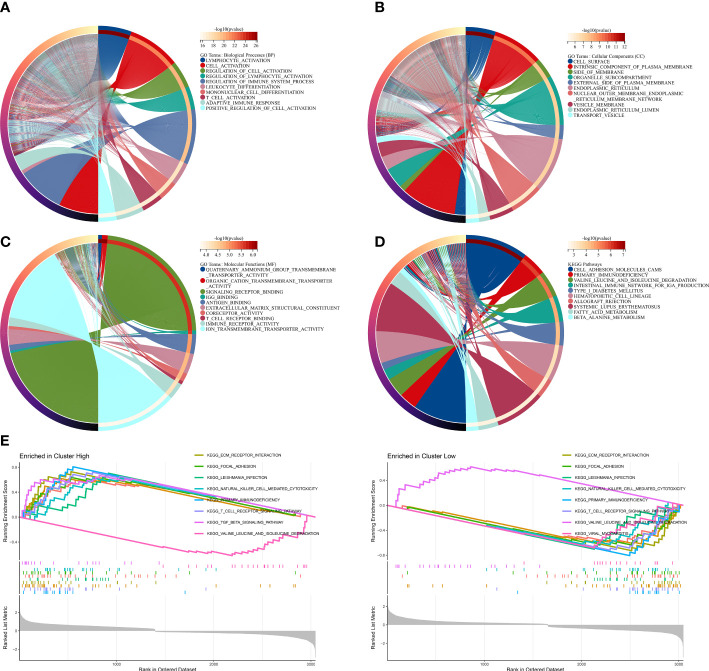
Functional enrichment analysis based on MRMGPS. **(A–C)** GO analysis based on MRMGPS-related DEGs between high- and low-risk groups. **(D)** KEGG analysis based on MRMGPS-related DEGs between high- and low-risk groups. **(E)** GSEA analysis based on MRMGPS-related DEGs between high- and low-risk groups.

To investigate potential biological functions and pathways related to MRMGPS in PCa, we screened out 4701 DEGs between two risk groups in the TCGA cohort. GO and KEGG analysis demonstrate that the DEGs were not only enriched in a variety of molecular functions and biological processes related to immunity, such as immune receptor activity, lymphocyte activation, and immune system regulation but also found that the primary immunodeficiency pathway was enriched (**Figures 7A–D**). In addition, GSEA analysis revealed that the high-risk group was mainly enriched in pathways about immune, including primary immunodeficiency, T cell receptor signaling pathway, and NK cell-mediated cytotoxicity (**Figure 7E**). These findings suggested that immunity might be a vital factor in bRFS disparities between subgroups.

### 3.8 Tumor immune microenvironment and genomic heterogeneity based on MRMGPS

**Figure 8:**
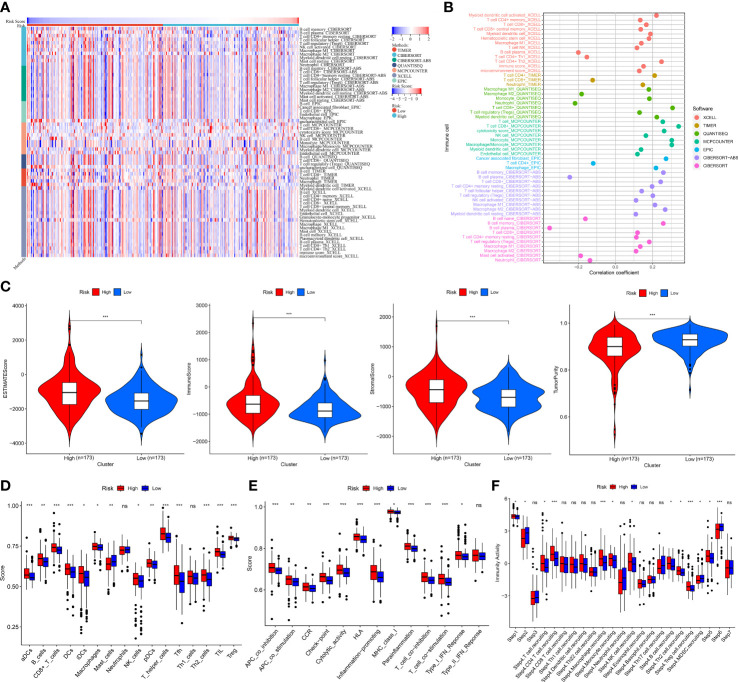
Tumor immune landscape based on MRMGPS. **(A)** Heatmap displaying enrichment of tumor immune-infiltrating cells through 7 algorithms between high- and low-risk groups. **(B)** Lollipop plot displaying the correlation coefficient of immune cells on the basis of risk score through 7 algorithms. **(C)** Comparison of the ESTIMATE scores, immune scores, stromal scores, and tumor purity between high- and low-risk groups. **(D)** Comparison of 16 cells related to immunity between high- and low-risk groups. **(E)** Comparison of 13 functions related to immunity between high- and low-risk groups. **(F)** Comparison of activity scores of each step in the cancer-immunity cycle between high- and low-risk groups. *P <0.05, **P < 0.01, ***P < 0.001.

To further study the relationship between MRMGPS and tumor immune microenvironment of the PCa samples, we first showed the heatmap and the correlation coefficient of the immune cells with risk score calculated by seven algorithms (**Figures 8A, B** **)**. Additionally, patients in the high-risk group had significantly higher stromal scores, ESTIMATE scores, and immune scores, but the tumor purity showed an inferior to that of the low-risk group (*P* < 0.001) (**Figure 8C**), indicating that PCa tissue from the high-risk subgroup contained more immune cells and immune molecules. It’s interesting to note that the high-risk group had much higher scores for 13 out of 16 of immune cells and 12 out of 13 of immune-related processes (**Figures 8D, E**). Furthermore, the immune activity scores at each step calculated by TIP analysis revealed that two groups had certain differences in 5 out of 7 steps of the cancer immune cycle (**Figure 8F**). These results revealed the unique tumor immune microenvironment in PCa.

**Figure 9:**
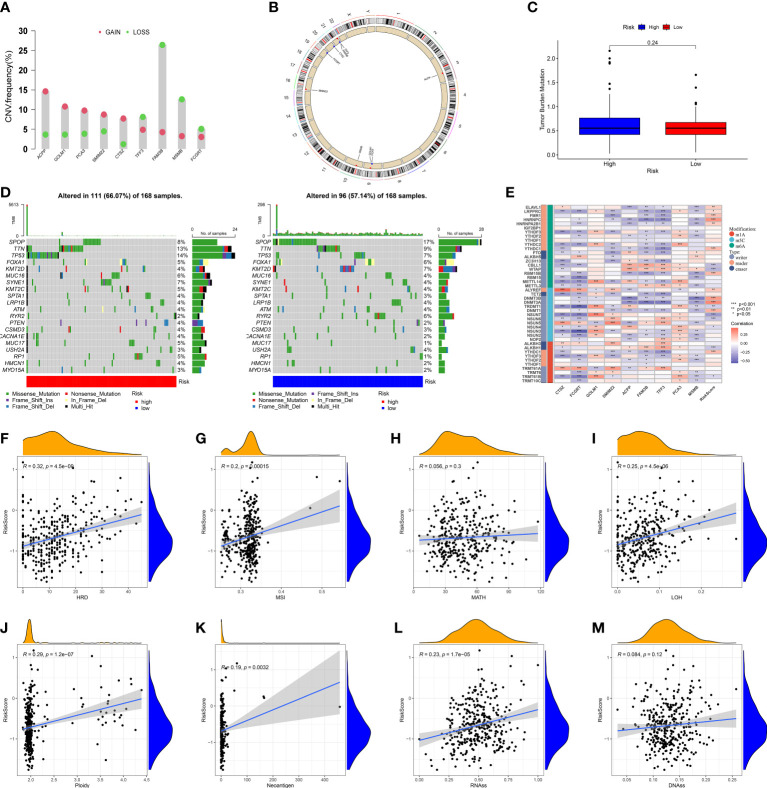
Genomic heterogeneity and tumor stemness based on MRMGPS. **(A)** CNV of the 9 MRMGPS-based genes. **(B)** Chromosomal location of CNV of MRMGPS-based genes. **(C)** Comparison of TMB between the low- and high-risk groups. **(D)** Waterfall plot displaying gene mutations in high- and low-risk groups. **(E–M)** Correlation between risk score and RNA-modified genes **(E)**, HDR **(F)**, MSI **(G)**, MATH **(H)**, LOH **(I)**, Ploidy **(J)**, Neoantigen **(K)**, RNAss **(L)**, DNAss**(M)**. CNV, copy number variation; TMB, tumor mutational burden; HRD, homologous recombination defect; MSI, microsatellite instability; MATH, mutant-allele tumor heterogeneity; LOH, loss of heterozygosity; RNAss, RNA-based stemness scores; DNAss, DNA-methylation-based stemness scores.

Given tumor heterogeneity has a profound impact on the immune microenvironment, we comprehensively explored crucial features of tumor heterogeneity based on MRMGPS including CNV, mutation profiles, TMB, epigenetic modification, HDR, MSI, MATH, LOH, ploidy, neoantigen, and tumor stemness (40, 41, 48).We found that among MRMGPS-based genes, *ACPP*, *GOLM1*, *PCA3*, *SMIM22*, and *CTSZ* showed comparatively high amplification, whereas *TFF3*, *FAM3B*, *MSMB*, and *FCGRT* showed primarily deletion (**Figures 9A, B**). However, no significant difference in TMB was found between the risk groups (*P* = 0.24). (**Figure 9C**). The mutation profiles showed that there were more mutations in the high-risk group, and the missense mutations in both groups were higher, in which the mutation rates of *TTN* and *TP53* were both higher than 10% (**Figure 9D**). Furthermore, the correlation between MRMGPS and epigenetic modification-related genes (m1A = 10, m5C = 13, and m6A = 20) was calculated and the result showed that 20 out of 43 genes were significantly related to risk score (**Figure 9E**). Moreover, we observed that risk score revealed a positive correlation with HRD (R = 0.32, *P* < 0.001), MSI (R = 0.20, *P* < 0.001), ploidy (R = 0.29, *P* < 0.001), neoantigen (R = 0.19, *P* < 0.01) and RNAss (R = 0.23, *P* < 0.001) but not with MATH and DNAss (**Figures 9F–M**). These results reflected the potential application value of MRMGPS in the exploration of heterogeneity of PCa.

### 3.9 Therapeutic responses and pharmaceutical analysis based on MRMGPS

**Figure 10:**
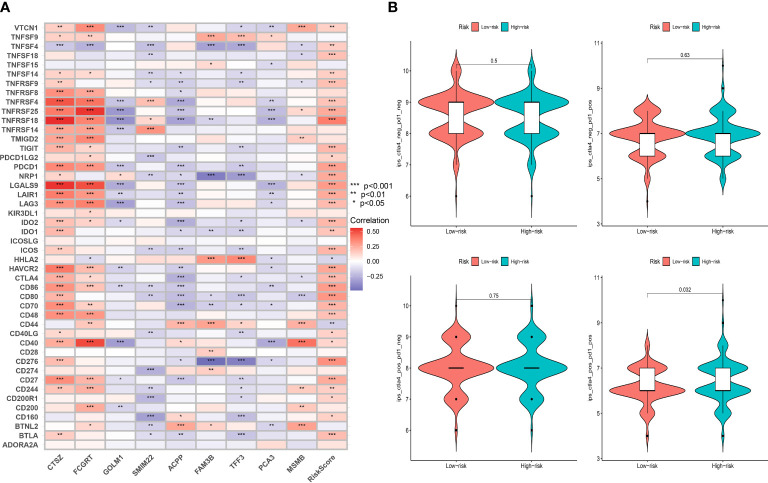
Evaluation of immunotherapeutic responses based on MRMGPS. **(A)** Correlation between immune checkpoint and MRMGPS-based genes. **(B)** Comparison of IPS of *PD1* and *CTLA4* between high- and low-risk groups. IPS, immunophenoscore.

Taking into account the critical role immune checkpoints played in immune responses and therapy, we further examined the relationship between the expression of immune checkpoints-associated genes and risk score. We discovered that 32 of 46 immune checkpoints, including *PDCD1* and *CTLA4*, showed a significant correlation with MRMGPS and 2 checkpoints, *HHLA2* and *CD44*, showed significant negative correlation (*P* < 0.05) (**Figure 10A**). Additionally, we specifically calculated the IPS of patients undergoing different treatments and showed that patients with low-risk score might be more responsive to *CTLA4*-positive/*PD1*-positive immunotherapy (*P* = 0.032) and had a more favorable therapeutic outcome (**Figure 10B**).

**Figure 11:**
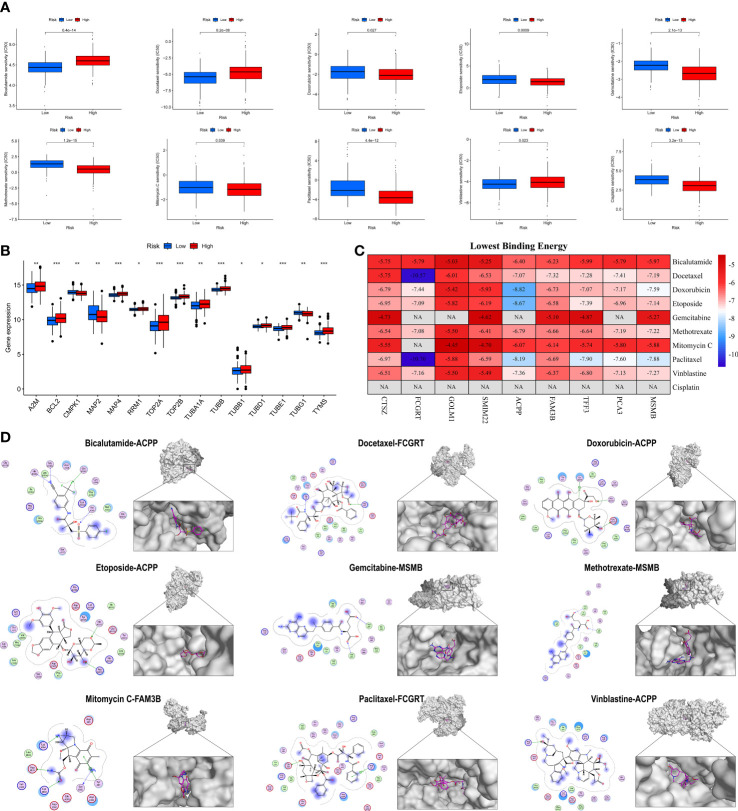
Evaluation of anti-PCa drugs based on MRMGPS. **(A)** Comparison of 10 anti-PCa drugs sensitivity (IC50) between high- and low-risk groups. **(B)** Comparison of the expression profile of 10 anti-PCa drugs targeted genes between high- and low-risk groups. **(C)** Heatmap displaying the lowest binding energy of 10 anti-PCa drugs docking with MRMGPS-based genes. **(D)** 2D (left) or 3D (right) binding mode of 10 anti-PCa drugs to the MRMGPS-based genes with the lowest binding energy. *P <0.05, **P < 0.01, ***P < 0.001.

To explore the possibility of applying MRMGPS to the personalized and accurate treatment of PCa, we first compared the differences in sensitivity to different anticancer drugs between high- and low-risk groups according to the IC50 value. The results showed that 10 commonly used anti-PCa drugs showed satisfactory differences in sensitivity, that is, patients in the high-risk group were more likely to be sensitive to bicalutamide and docetaxel, whereas individuals in the low-risk group were more likely to be sensitive to doxorubicin, etoposide, gemcitabine, methotrexate, mitomycin C, paclitaxel, vinblastine, and cisplatin (**Figure 11A**). Based on the targeted genes obtained from the DrugBank database (**Table S7**), we further found that 15 genes targeted by these drugs had increased expression in the high-risk group (**Figure 11B**). The above evidence indicated that MRMGPS might help clinicians to select personalized drugs and treatments according to different patients.

We further evaluated the affinity of targeted drugs to genes and used MOE for molecular docking to explore the internal relationship between MRMGPS and 10 anti-PCa drugs. The results demonstrated that 51 out of 75 minimum binding energy of drug-gene pairs were less than -6.0 kcal/mol, except that cisplatin could not be docked (**Figures 11C, D**), indicating that the majority of targeted drugs and MRMGPS-based genes maintained extreme stability of the intersectional combination. These findings revealed that there might be a certain relationship between genes and drugs, which provided a certain theoretical value for the study of drugs for PCa.

## 4 Discussion

Due to the rapid development of scRNA-seq technologies, researchers obtained unprecedented opportunities to simultaneously assess thousands of cells within a sample, enabling the complex molecular features of the TME to be revealed (18). However, the majority of recent research concentrated on adaptive immune cells in TME, and the contribution of innate immune cells that may have a significant impact on prognosis and treatment response in certain cancers has not yet gotten adequate attention (8, 9). TAMs represent the primary element of the innate immune system in TME, which are the most abundant infiltrating immune cells and constitute up to 50% of the cell mass of human tumors (49). Increasing studies reveal that the prognosis of PCa patients is closely correlated with the abundance of tumor-infiltrating macrophages now (50–53). Given the critical role of macrophages in the immune microenvironment and prognostic prediction, we attempted to identify the MRMGs associated with the prognosis of PCa patients. To our knowledge, this is the first study on mining MRMGs through scRNA-seq data to construct a signature to predict prognosis, analyze TME, and select drugs in PCa.

In this study, we first performed scRNA-seq analysis and systematically investigated the 307 MRMGs. Three clusters were identified based on the expression of MRMGs in TCGA and there were significant discrepancies in clinicopathological characteristics, somatic mutations, and immune phenotypes between each cluster. These results demonstrated that macrophages had a significant role in the development and progression of PCa. Subsequently, we established MRMGPS based on the MRMGs in the TCGA cohort and verified its excellent prognostic performance in a merged GEO validation cohort. The results also showed that MRMGPS could function as a solitary prognostic factor.

The majority of MRMGPS-based genes (*CTSZ*, *FCGRT*, *GOLM1*, *SMIM22*, *ACPP*, *FAM3B*, *TFF3*, *PCA3*, and *MSMB*) are associated with either TAMs activity or the prognosis of PCa patients. *CTSZ* is an immune-related gene also known as *CTSX*, which is consistently expressed by immune cells, especially TAMs (54, 55). The high expression level of *CTSZ*, found in prostatic intraepithelial neoplasia and PCa, may contribute a significant role in the early tumorigenesis of PCa (55), which is further clarified by RT-qPCR. *FCGRT* is also known as *FcRn*, which encodes a receptor that binds the Fc region of monomeric immunoglobulin G. Previous studies reveal that *FCGR*T plays a crucial role in anti-tumor immunosurveillance (56) and lack of *FCGRT* could impair development and function of innate immune cells leading to poor prognosis of cancer (57). *GOLM1*, known as *GP73* or *GOLPH2* and overexpressed in multicancer including PCa, plays a crucial role in the tumor immune microenvironment (58, 59). A previous study reveals that overexpressed *GOLM1* promotes PD-L1 transport into TAMs in hepatocellular carcinoma, aggravating CD8+ T cell suppression and promoting tumor progression (60), which is similar to our results on MRMGPS-based immune cell infiltration in PCa. *SMIM22* also called *CASIMO*1 is a critical factor for proliferation and cell cycle progression in breast cancer (61). *TFF3* is highly expressed in PCa and enhances the carcinogenic properties of PCa cells (62, 63). As for *PCA3* and *MSMB*, it has been reported that they are specificity biomarkers and helpful for the diagnosis of PCa (64–66). Although there are few studies focused on the influence of *ACPP* and *FAM3B* in cancer, we believe that the potential mechanisms of all MRMGPS-based genes in tumorigenesis and progression deserve further exploration combined with the results of our analysis.

As expected, our functional analysis results showed that MRMGPS was mainly embodied in immune-related biological processes and pathways. Moreover, immune cells infiltrated in the TME play a critical role in tumor development and prognosis (67). Therefore, it is so critical to study the landscape of MRMGPS in TME. Our result suggested that differences in the infiltration of the immune-related cells and functions between MRMGPS-based groups, such as high-risk group with more immunosuppressive cells like pCDs and Tregs, might explain the poor prognosis of patients in high-risk group. As we explored the features of genomic heterogeneity and tumor stemness that influence the immune microenvironment, we found that TMB did not show a significant difference between two risk groups, which was similar to the results of a previous prospective study of PCa (68). Besides mutations in overall genes, TMB has been linked to immunotherapy responses, particularly ICI therapy, of which *CTLA4* and *PD1* are commonly targeted molecules (69). However, if patients are not differentiated by specific markers, the efficacy of extensive treatment is not well for PCa (70). Interestingly, our research found that immunotherapy for high-risk patients with *PD1*-positive and *CTLA4*-positive would achieve more satisfactory results. A previous prospective study on PCa immunotherapy found that the expression of *VISTA* in TAM restricted the compensation pathway of ICI treatment efficiency (71), which may well explain the interesting phenomenon we found. The above results and our drug analysis based on MRMGPS mean that MRMGPS is of great significance in personalized and precise drug use for PCa patients.

Although our findings are so exciting, there are some limitations to this study that need to be taken into consideration. First of all, except for the basic verification experiments, the rest of the data in this study come from public databases, and the experimental study of the functional mechanism is also crucial. Finally, this study is based on a retrospective analysis of the database, and the risk model we established needs further support from prospective research evidence.

## 5 Conclusions

In summary, through multi-omics analysis, we established a prognostic signature based on MRMGs with excellent performance for prediction, tumor immune research, and drug selection. Additionally, MRMGPS is not only a potential biomarker for evaluating bRFS rate and immune characteristics, but it also provides useful insights and a theoretical basis for creating personalized and accurate treatment strategies and drug decisions for patients with PCa.

## Data availability statement

Publicly available datasets were analyzed in this study. This data can be found here: https://portal.gdc.cancer.gov/ and https://www.ncbi.nlm.nih.gov/.

## Ethics statement

The studies involving human participants were reviewed and approved by Clinical Ethics Board of the Third Affiliated Hospital of Sun Yat-sen University (Guangzhou, China). The patients/participants provided their written informed consent to participate in this study.

## Author contributions

WZ: Conceptualization, Investigation, Methodology, Writing - Review & Editing. JW and JH: Writing - Original Draft, Resources, Validation. DX and FL: Software, Formal analysis. CW and HZ: Data curation. JZ: Visualization. XL: Funding acquisition, Writing - Review & Editing. WL and XW: Supervision, Project administration, Funding acquisition, Writing - Review & Editing. All authors have read and approved the final manuscript.

## References

[1] Siegel RL Miller KD Fuchs HE Jemal A. Cancer statistics, 2022. CA Cancer J Clin (2022) 72(1):7–33. doi: 10.3322/caac.21708 35020204

[2] Teo MY Rathkopf DE Kantoff P. Treatment of advanced prostate cancer. Annu Rev Med (2019) 70:479–99. doi: 10.1146/annurev-med-051517-011947 PMC6441973 30691365

[3] Sandhu S Moore CM Chiong E Beltran H Bristow RG Williams SG. Prostate cancer. Lancet (2021) 398(10305):1075–90. doi: 10.1016/s0140-6736(21)00950-8 34370973

[4] Drake CG. Prostate cancer as a model for tumor immunotherapy. Nat Rev Immunol (2010) 10(8):580–93. doi: 10.1038/nri2817 PMC3082366 20651745

[5] Bilusic M Madan RA Gulley JL. Immunotherapy of prostate cancer: facts and hopes. Clin Cancer Res (2017) 23(22):6764–70. doi: 10.1158/1078-0432.CCR-17-0019 PMC5690854 28663235

[6] Kgatle MM Boshomane TMG Lawal IO Mokoala KMG Mokgoro NP Lourens N. Immune checkpoints, inhibitors and radionuclides in prostate cancer: promising combinatorial therapy approach. Int J Mol Sci (2021) 22(8):4109. doi: 10.3390/ijms22084109 33921181 PMC8071559

[7] Jin MZ Jin WL. The updated landscape of tumor microenvironment and drug repurposing. Signal Transduct Target Ther (2020) 5(1):166. doi: 10.1038/s41392-020-00280-x 32843638 PMC7447642

[8] Belli C Trapani D Viale G D'Amico P Duso BA Della Vigna P. Targeting the microenvironment in solid tumors. Cancer Treat Rev (2018) 65:22–32. doi: 10.1016/j.ctrv.2018.02.004 29502037

[9] Xiao Y Yu D. Tumor microenvironment as a therapeutic target in cancer. Pharmacol Ther (2021) 221:107753. doi: 10.1016/j.pharmthera.2020.107753 33259885 PMC8084948

[10] Chen F Zhuang X Lin L Yu P Wang Y Shi Y. New horizons in tumor microenvironment biology: challenges and opportunities. BMC Med (2015) 13:45. doi: 10.1186/s12916-015-0278-7 25857315 PMC4350882

[11] Vitale I Manic G Coussens LM Kroemer G Galluzzi L. Macrophages and metabolism in the tumor microenvironment. Cell Metab (2019) 30(1):36–50. doi: 10.1016/j.cmet.2019.06.001 31269428

[12] Varol C Mildner A Jung S. Macrophages: development and tissue specialization. Annu Rev Immunol (2015) 33:643–75. doi: 10.1146/annurev-immunol-032414-112220 25861979

[13] Anderson NR Minutolo NG Gill S Klichinsky M. Macrophage-based approaches for cancer immunotherapy. Cancer Res (2021) 81(5):1201–8. doi: 10.1158/0008-5472.Can-20-2990 33203697

[14] Yang L Zhang Y. Tumor-associated macrophages: from basic research to clinical application. J Hematol Oncol (2017) 10(1):58. doi: 10.1186/s13045-017-0430-2 28241846 PMC5329931

[15] Masetti M Carriero R Portale F Marelli G Morina N Pandini M. Lipid-loaded tumor-associated macrophages sustain tumor growth and invasiveness in prostate cancer. J Exp Med (2022) 219(2):e20210564. doi: 10.1084/jem.20210564 34919143 PMC8932635

[16] Huang R Wang S Wang N Zheng Y Zhou J Yang B. CCL5 derived from tumor-associated macrophages promotes prostate cancer stem cells and metastasis *via* activating β-catenin/STAT3 signaling. Cell Death Dis (2020) 11(4):234. doi: 10.1038/s41419-020-2435-y 32300100 PMC7162982

[17] Cioni B Zaalberg A van Beijnum JR Melis MHM van Burgsteden J Muraro MJ. Androgen receptor signalling in macrophages promotes TREM-1-mediated prostate cancer cell line migration and invasion. Nat Commun (2020) 11(1):4498. doi: 10.1038/s41467-020-18313-y 32908142 PMC7481219

[18] Kumar MP Du J Lagoudas G Jiao Y Sawyer A Drummond DC. Analysis of single-cell RNA-seq identifies cell-cell communication associated with tumor characteristics. Cell Rep (2018) 25(6):1458–1468.e4. doi: 10.1016/j.celrep.2018.10.047 30404002 PMC7009724

[19] Wu T Wu X Wang HY Chen L. Immune contexture defined by single cell technology for prognosis prediction and immunotherapy guidance in cancer. Cancer Commun (Lond) (2019) 39(1):21. doi: 10.1186/s40880-019-0365-9 30999966 PMC6471962

[20] Chen S Zhu G Yang Y Wang F Xiao YT Zhang N. Single-cell analysis reveals transcriptomic remodellings in distinct cell types that contribute to human prostate cancer progression. Nat Cell Biol (2021) 23(1):87–98. doi: 10.1038/s41556-020-00613-6 33420488

[21] Song H Weinstein HNW Allegakoen P Wadsworth MH Xie J Yang H. Single-cell analysis of human primary prostate cancer reveals the heterogeneity of tumor-associated epithelial cell states. Nat Commun (2022) 13(1):141. doi: 10.1038/s41467-021-27322-4 35013146 PMC8748675

[22] Ge G Han Y Zhang J Li X Liu X Gong Y. Single-cell RNA-seq reveals a developmental hierarchy super-imposed over subclonal evolution in the cellular ecosystem of prostate cancer. Adv Sci (Weinh) (2022) 9(15):e2105530. doi: 10.1002/advs.202105530 35322584 PMC9131431

[23] Johnson WE Li C Rabinovic A. Adjusting batch effects in microarray expression data using empirical bayes methods. Biostatistics (2007) 8(1):118–27. doi: 10.1093/biostatistics/kxj037 16632515

[24] Charoentong P Finotello F Angelova M Mayer C Efremova M Rieder D. Pan-cancer immunogenomic analyses reveal genotype-immunophenotype relationships and predictors of response to checkpoint blockade. Cell Rep (2017) 18(1):248–62. doi: 10.1016/j.celrep.2016.12.019 28052254

[25] Liao Y Wang J Jaehnig EJ Shi Z Zhang B. WebGestalt 2019: gene set analysis toolkit with revamped UIs and APIs. Nucleic Acids Res (2019) 47(W1):W199–205. doi: 10.1093/nar/gkz401 PMC6602449 31114916

[26] Szklarczyk D Gable AL Lyon D Junge A Wyder S Huerta-Cepas J. STRING v11: protein-protein association networks with increased coverage, supporting functional discovery in genome-wide experimental datasets. Nucleic Acids Res (2019) 47(D1):D607–13. doi: 10.1093/nar/gky1131 PMC6323986 30476243

[27] Shannon P Markiel A Ozier O Baliga NS Wang JT Ramage D. Cytoscape: a software environment for integrated models of biomolecular interaction networks. Genome Res (2003) 13(11):2498–504. doi: 10.1101/gr.1239303 PMC403769 14597658

[28] Sun Y Xu Z Jiang J Xu T Xu J Liu P. High expression of succinate dehydrogenase subunit a which is regulated by histone acetylation, acts as a good prognostic factor of multiple myeloma patients. Front Oncol (2020) 10:563666. doi: 10.3389/fonc.2020.563666 33014881 PMC7511799

[29] Asplund A Edqvist PH Schwenk JM Pontén F. Antibodies for profiling the human proteome-the human protein atlas as a resource for cancer research. Proteomics (2012) 12(13):2067–77. doi: 10.1002/pmic.201100504 22623277

[30] Racle J Gfeller D. EPIC: a tool to estimate the proportions of different cell tpes from bulk gene expression data. Methods Mol Biol (2020) 2120:233–48. doi: 10.1007/978-1-0716-0327-7_17 32124324

[31] Aran D Hu Z Butte AJ. xCell: digitally portraying the tissue cellular heterogeneity landscape. Genome Biol (2017) 18(1):220. doi: 10.1186/s13059-017-1349-1 29141660 PMC5688663

[32] Becht E Giraldo NA Lacroix L Buttard B Elarouci N Petitprez F. Estimating the population abundance of tissue-infiltrating immune and stromal cell populations using gene expression. Genome Biol (2016) 17(1):218. doi: 10.1186/s13059-016-1070-5 27765066 PMC5073889

[33] Finotello F Mayer C Plattner C Laschober G Rieder D Hackl H. Molecular and pharmacological modulators of the tumor immune contexture revealed by deconvolution of RNA-seq data. Genome Med (2019) 11(1):34. doi: 10.1186/s13073-019-0638-6 31126321 PMC6534875

[34] Newman AM Liu CL Green MR Gentles AJ Feng W Xu Y. Robust enumeration of cell subsets from tissue expression profiles. Nat Methods (2015) 12(5):453–7. doi: 10.1038/nmeth.3337 PMC4739640 25822800

[35] Li T Fan J Wang B Traugh N Chen Q Liu JS. TIMER: a web server for comprehensive analysis of tumor-infiltrating immune cells. Cancer Res (2017) 77(21):e108–10. doi: 10.1158/0008-5472.Can-17-0307 PMC6042652 29092952

[36] Bindea G Mlecnik B Tosolini M Kirilovsky A Waldner M Obenauf AC. Spatiotemporal dynamics of intratumoral immune cells reveal the immune landscape in human cancer. Immunity (2013) 39(4):782–95. doi: 10.1016/j.immuni.2013.10.003 24138885

[37] Rooney MS Shukla SA Wu CJ Getz G Hacohen N. Molecular and genetic properties of tumors associated with local immune cytolytic activity. Cell (2015) 160(1-2):48–61. doi: 10.1016/j.cell.2014.12.033 25594174 PMC4856474

[38] Chen X Chen H Yao H Zhao K Zhang Y He D. Turning up the heat on non-immunoreactive tumors: pyroptosis influences the tumor immune microenvironment in bladder cancer. Oncogene (2021) 40(45):6381–93. doi: 10.1038/s41388-021-02024-9 34588621

[39] Mermel CH Schumacher SE Hill B Meyerson ML Beroukhim R Getz G. Gistic2.0 facilitates sensitive and confident localization of the targets of focal somatic copy-number alteration in human cancers. Genome Biol (2011) 12(4):R41. doi: 10.1186/gb-2011-12-4-r41 21527027 PMC3218867

[40] Bonneville R Krook MA Kautto EA Miya J Wing MR Chen HZ. Landscape of microsatellite instability across 39 cancer types. JCO Precis Oncol (2017) 2017:PO.17.00073. doi: 10.1200/po.17.00073 29850653 PMC5972025

[41] Malta TM Sokolov A Gentles AJ Burzykowski T Poisson L Weinstein JN. Machine learning identifies stemness features associated with oncogenic dedifferentiation. Cell (2018) 173(2):338–354.e15. doi: 10.1016/j.cell.2018.03.034 29625051 PMC5902191

[42] Thorsson V Gibbs DL Brown SD Wolf D Bortone DS Ou Yang TH. The immune landscape of cancer. Immunity (2018) 48(4):812–830.e14. doi: 10.1016/j.immuni.2018.03.023 29628290 PMC5982584

[43] Yang W Soares J Greninger P Edelman EJ Lightfoot H Forbes S. Genomics of drug sensitivity in cancer (GDSC): a resource for therapeutic biomarker discovery in cancer cells. Nucleic Acids Res (2013) 41(Database issue):D955–61. doi: 10.1093/nar/gks1111 PMC3531057 23180760

[44] Wishart DS Feunang YD Guo AC Lo EJ Marcu A Grant JR. DrugBank 5.0: a major update to the DrugBank database for 2018. Nucleic Acids Res (2018) 46(D1):D1074–82. doi: 10.1093/nar/gkx1037 PMC5753335 29126136

[45] Wang Y Bryant SH Cheng T Wang J Gindulyte A Shoemaker BA. Pubchem bioassay: 2017 update. Nucleic Acids Res (2017) 45(D1):D955–63. doi: 10.1093/nar/gkw1118 PMC5210581 27899599

[46] Rose PW Prlić A Altunkaya A Bi C Bradley AR Christie CH. The RCSB protein data bank: integrative view of protein, gene and 3D structural information. Nucleic Acids Res (2017) 45(D1):D271–81. doi: 10.1093/nar/gkw1000 PMC5210513 27794042

[47] Adams LA Möller M Nebel A Schreiber S van der Merwe L van Helden PD. Polymorphisms in MC3R promoter and CTSZ 3'UTR are associated with tuberculosis susceptibility. Eur J Hum Genet (2011) 19(6):676–81. doi: 10.1038/ejhg.2011.1 PMC3110050 21368909

[48] Fraser M Sabelnykova VY Yamaguchi TN Heisler LE Livingstone J Huang V. Genomic hallmarks of localized, non-indolent prostate cancer. Nature (2017) 541(7637):359–64. doi: 10.1038/nature20788 28068672

[49] Cao J Liu J Xu R Zhu X Zhao X Qian BZ. Prognostic role of tumour-associated macrophages and macrophage scavenger receptor 1 in prostate cancer: a systematic review and meta-analysis. Oncotarget (2017) 8(47):83261–9. doi: 10.18632/oncotarget.18743 PMC5669966 29137340

[50] Shimura S Yang G Ebara S Wheeler TM Frolov A Thompson TC. Reduced infiltration of tumor-associated macrophages in human prostate cancer: association with cancer progression. Cancer Res (2000) 60(20):5857–61. 11059783

[51] Ok Atılgan A Özdemir BH Akçay EY Ataol Demirkan Ö Tekindal MA Özkardeş H. Role of tumor-associated macrophages in the Hexim1 and TGFβ/SMAD pathway, and their influence on progression of prostatic adenocarcinoma. Pathol Res Pract (2016) 212(2):83–92. doi: 10.1016/j.prp.2015.10.011 26608417

[52] Takayama H Nonomura N Nishimura K Oka D Shiba M Nakai Y. Decreased immunostaining for macrophage scavenger receptor is associated with poor prognosis of prostate cancer. BJU Int (2009) 103(4):470–4. doi: 10.1111/j.1464-410X.2008.08013.x 18778349

[53] Lissbrant IF Stattin P Wikstrom P Damber JE Egevad L Bergh A. Tumor associated macrophages in human prostate cancer: relation to clinicopathological variables and survival. Int J Oncol (2000) 17(3):445–51. doi: 10.3892/ijo.17.3.445 10938382

[54] Jechorek D Votapek J Meyer F Kandulski A Roessner A Franke S. Characterization of cathepsin X in colorectal cancer development and progression. Pathol Res Pract (2014) 210(12):822–9. doi: 10.1016/j.prp.2014.08.014 25442015

[55] Nägler DK Krüger S Kellner A Ziomek E Menard R Buhtz P. Up-regulation of cathepsin X in prostate cancer and prostatic intraepithelial neoplasia. Prostate (2004) 60(2):109–19. doi: 10.1002/pros.20046 15162377

[56] Baker K Rath T Flak MB Arthur JC Chen Z Glickman JN. Neonatal fc receptor expression in dendritic cells mediates protective immunity against colorectal cancer. Immunity (2013) 39(6):1095–107. doi: 10.1016/j.immuni.2013.11.003 PMC3902970 24290911

[57] Castaneda DC Dhommée C Baranek T Dalloneau E Lajoie L Valayer A. Lack of FcRn impairs natural killer cell development and functions in the tumor microenvironment. Front Immunol (2018) 9:2259. doi: 10.3389/fimmu.2018.02259 30323819 PMC6172308

[58] Kristiansen G Fritzsche FR Wassermann K Jäger C Tölls A Lein M. GOLPH2 protein expression as a novel tissue biomarker for prostate cancer: implications for tissue-based diagnostics. Br J Cancer (2008) 99(6):939–48. doi: 10.1038/sj.bjc.6604614 PMC2538754 18781151

[59] Varambally S Laxman B Mehra R Cao Q Dhanasekaran SM Tomlins SA. Golgi protein GOLM1 is a tissue and urine biomarker of prostate cancer. Neoplasia (2008) 10(11):1285–94. doi: 10.1593/neo.08922 PMC2570605 18953438

[60] Chen J Lin Z Liu L Zhang R Geng Y Fan M. GOLM1 exacerbates CD8^+^ T cell suppression in hepatocellular carcinoma by promoting exosomal PD-L1 transport into tumor-associated macrophages. Signal Transduct Target Ther (2021) 6(1):397. doi: 10.1038/s41392-021-00784-0 34795203 PMC8602261

[61] Polycarpou-Schwarz M Groß M Mestdagh P Schott J Grund SE Hildenbrand C. The cancer-associated microprotein CASIMO1 controls cell proliferation and interacts with squalene epoxidase modulating lipid droplet formation. Oncogene (2018) 37(34):4750–68. doi: 10.1038/s41388-018-0281-5 29765154

[62] Perera O Evans A Pertziger M MacDonald C Chen H Liu D-X. Trefoil factor 3 (TFF3) enhances the oncogenic characteristics of prostate carcinoma cells and reduces sensitivity to ionising radiation. Cancer Lett (2015) 361(1):104–11. doi: 10.1016/j.canlet.2015.02.051 25748388

[63] Garraway IP Seligson D Said J Horvath S Reiter RE. Trefoil factor 3 is overexpressed in human prostate cancer. Prostate (2004) 61(3):209–14. doi: 10.1002/pros.20096 15368472

[64] Gunelli R Fragalà E Fiori M. PCA3 in prostate cancer. Methods Mol Biol (2021) 2292:105–13. doi: 10.1007/978-1-0716-1354-2_9 33651355

[65] Hessels D Schalken JA. The use of PCA3 in the diagnosis of prostate cancer. Nat Rev Urol (2009) 6(5):255–61. doi: 10.1038/nrurol.2009.40 19424173

[66] Sutcliffe S De Marzo AM Sfanos KS Laurence M. MSMB variation and prostate cancer risk: clues towards a possible fungal etiology. Prostate (2014) 74(6):569–78. doi: 10.1002/pros.22778 PMC4037912 24464504

[67] Barnes TA Amir E. Hype or hope: the prognostic value of infiltrating immune cells in cancer. Br J Cancer (2017) 117(4):451–60. doi: 10.1038/bjc.2017.220 PMC5558691 28704840

[68] McGrail DJ Pilié PG Rashid NU Voorwerk L Slagter M Kok M. High tumor mutation burden fails to predict immune checkpoint blockade response across all cancer types. Ann Oncol (2021) 32(5):661–72. doi: 10.1016/j.annonc.2021.02.006 PMC8053682 33736924

[69] Chan TA Yarchoan M Jaffee E Swanton C Quezada SA Stenzinger A. Development of tumor mutation burden as an immunotherapy biomarker: utility for the oncology clinic. Ann Oncol (2019) 30(1):44–56. doi: 10.1093/annonc/mdy495 30395155 PMC6336005

[70] Vitkin N Nersesian S Siemens DR Koti M. The tumor immune contexture of prostate cancer. Front Immunol (2019) 10:603. doi: 10.3389/fimmu.2019.00603 30984182 PMC6447686

[71] Gao J Ward JF Pettaway CA Shi LZ Subudhi SK Vence LM. Vista is an inhibitory immune checkpoint that is increased after ipilimumab therapy in patients with prostate cancer. Nat Med (2017) 23(5):551–5. doi: 10.1038/nm.4308 PMC5466900 28346412

