# Supplementary material for: Multi-omics analysis reveals a macrophage-related marker gene signature for prognostic prediction, immune landscape, genomic heterogeneity, and drug choices in prostate cancer
Source: Front Immunol. 2023 Apr 14;14:1122670. doi: 10.3389/fimmu.2023.1122670 (PMC10140525; doi:10.3389/fimmu.2023.1122670)
Supplement: Supplementary file 1 [file DataSheet_1.docx]

Supplementary Material

Multi-Omics Analysis Reveals a Macrophage-Related Marker Gene Signature for Prognostic Prediction, Immune Landscape, Genomic Heterogeneity, and Drug Choices in Prostate Cancer

Weian Zhu†, Jianjie Wu†, Jiongduan Huang†, Dongming Xiao, Fengao Li, Chenglun Wu, Xiaojuan Li, Hengda Zeng, Jiayu Zheng, Wenjie Lai*, Xingqiao Wen*

*** Correspondence:** Xingqiao Wen: [wenxq@mail.sysu.edu.cn](mailto:wenxq@mail.sysu.edu.cn); Wenjie Lai: jie_small@163.com

# Supplementary Figures and Tables

## Supplementary Figures


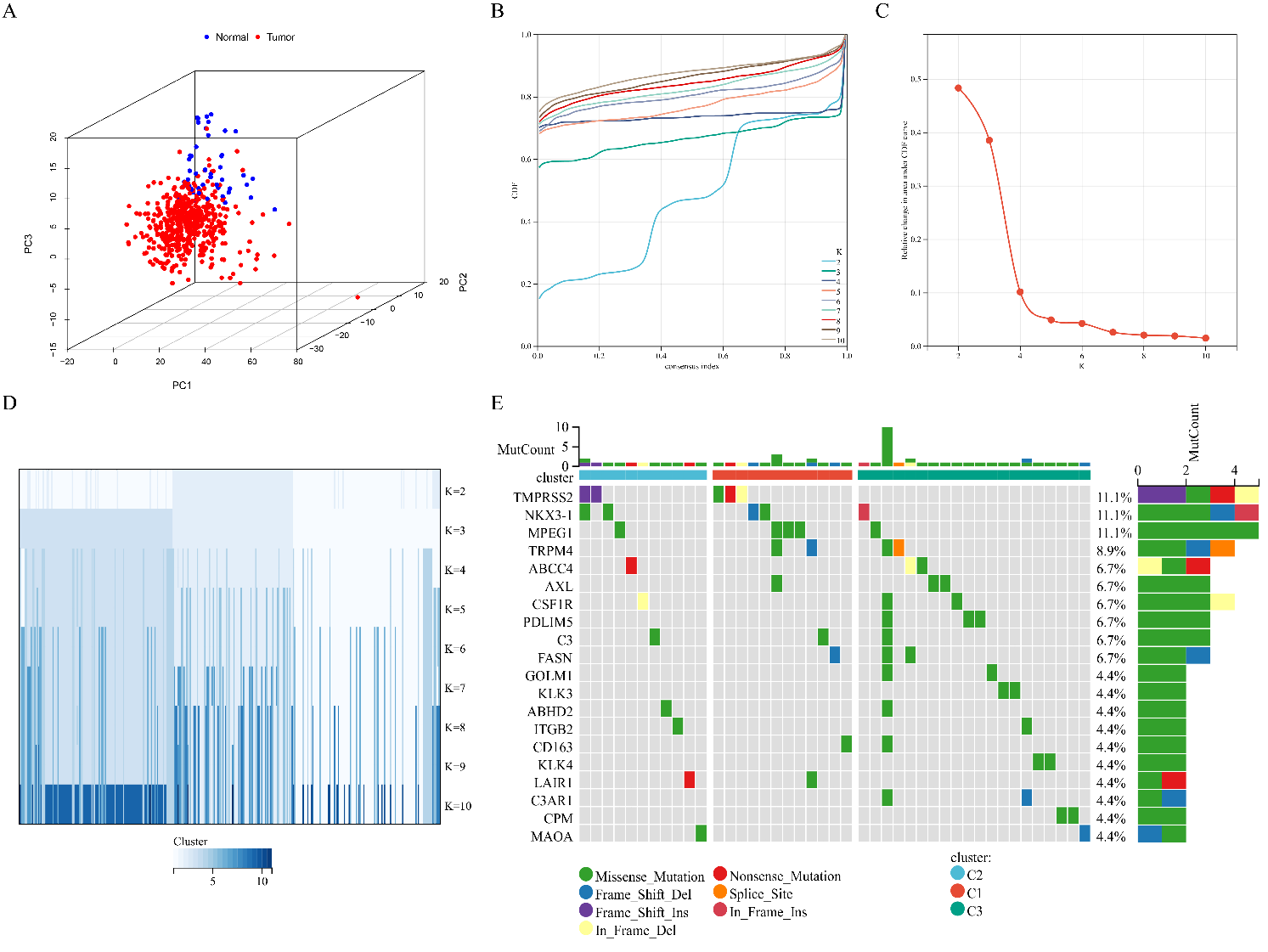


**Supplementary Figure 1.** **Consensus clustering and mutation analysis of 307 MRMGs.** **(A)** 3D-PCA plot displaying the distribution between PCa and normal samples based on 307 MRMGs. **(B)** CDF curves with different values of k. **(C)** Relative change in area under CDF curves with different values of k. **(D)** Sample distribution with different values of k. **(E)** Waterfall plot displaying gene mutations in three clusters (C1=121, C2=99, C3=126). MRMGs, macrophage-related marker genes; PCA, principal component analysis; CDF, cumulative distribution function.


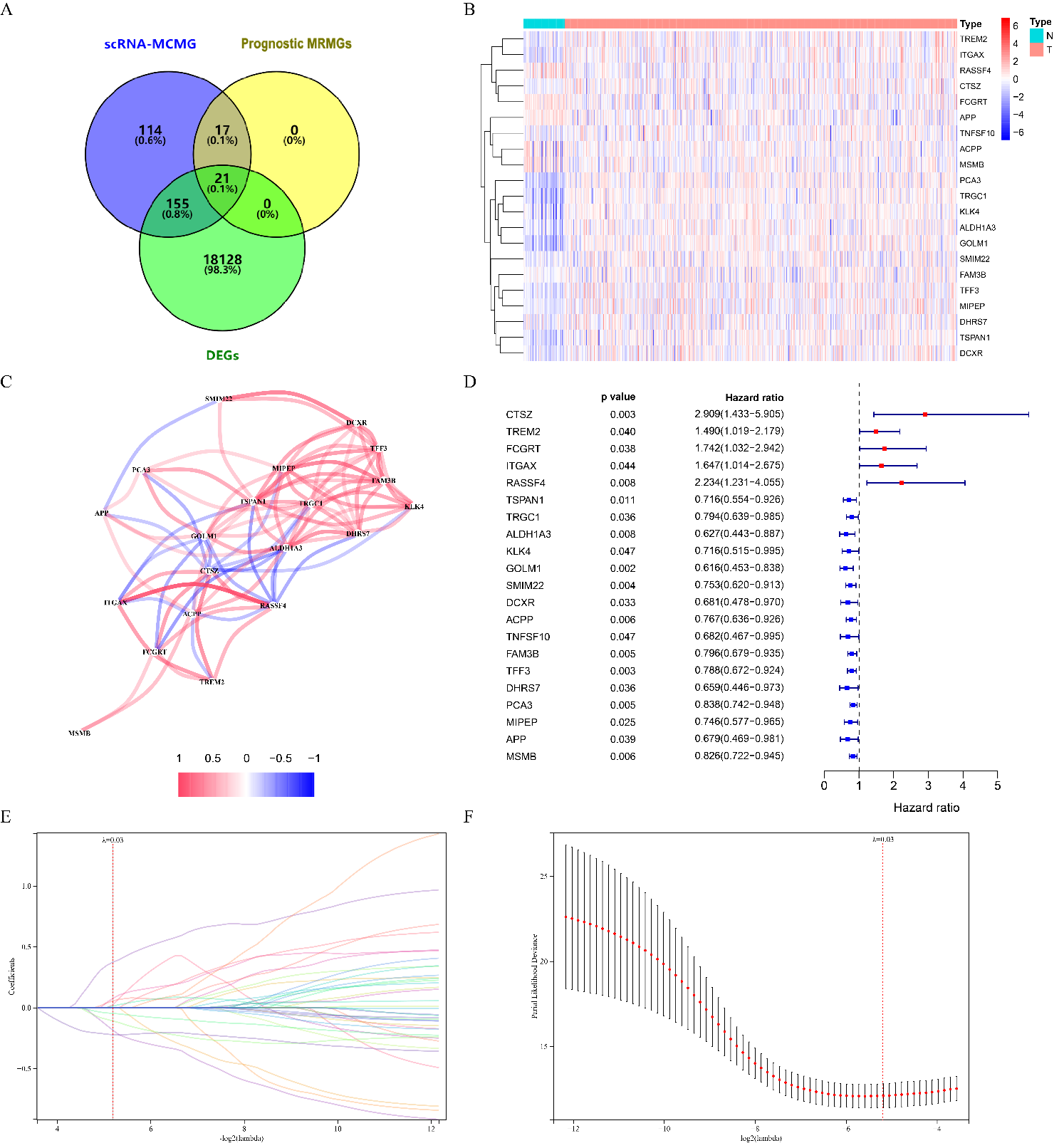


**Supplementary Figure 2. Construction of an MRMGPS in TCGA cohort.** **(A)** Venn diagram displaying the 21 overlapping genes of 307 scRNA-MRMGs, 18394 DEGs between PCa and normal samples, and 38 prognostic MRMGs. **(B)** Heatmap displaying expression profile of the 21 overlapping genes in PCa and normal samples. **(C)** Correlation among 21 overlapping genes. **(D)** Forest diagram displaying hazard ratio of 21 overlapping genes in univariate Cox regression analysis. **(E)** LASSO coefficient curve of different quantitative variables. **(F)** Partial likelihood deviance of the best parameter (λ). TCGA, The Cancer Genome Atlas; MRMGPS, macrophage-related marker gene prognostic signature; DEGs, differentially expressed genes; LASSO, least absolute shrinkage and selection operator.


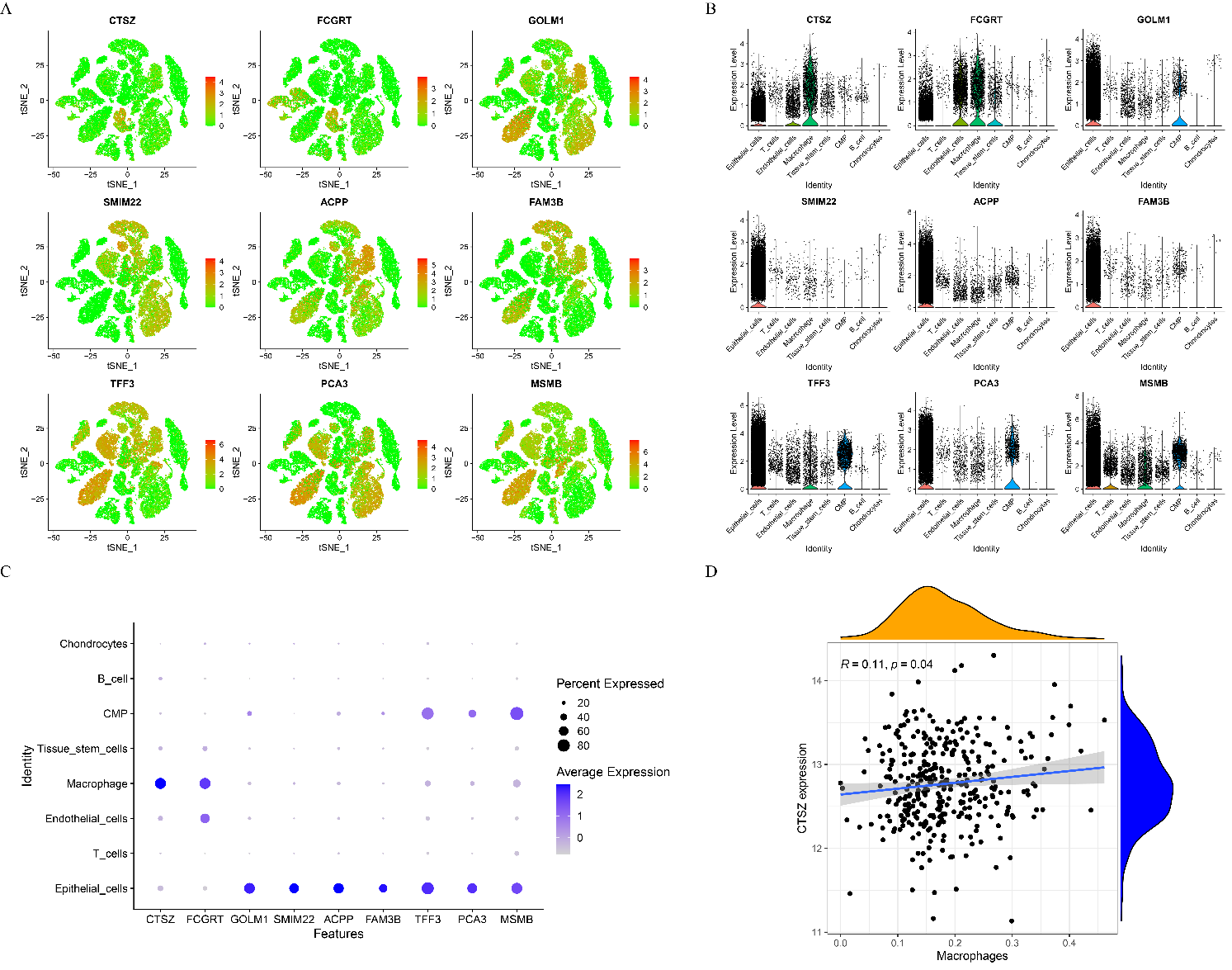


**Supplementary Figure 3. The expression profiles of CTSZ at scRNA-seq and bulk RNA-seq level.** **(A-C)** t-SNE plots **(A)**, bubble plots **(B)**, and violin plots **(C)** displaying the distribution of MRMGPS-based genes in the scRNA-seq data. **(D)** The correlation between CTSZ expression and macrophage proportions deconvoluted from the bulk RNA-seq data of TCGA cohort. t-SNE, t-distributed stochastic neighbor embedding; CMP, common myeloid progenitors.


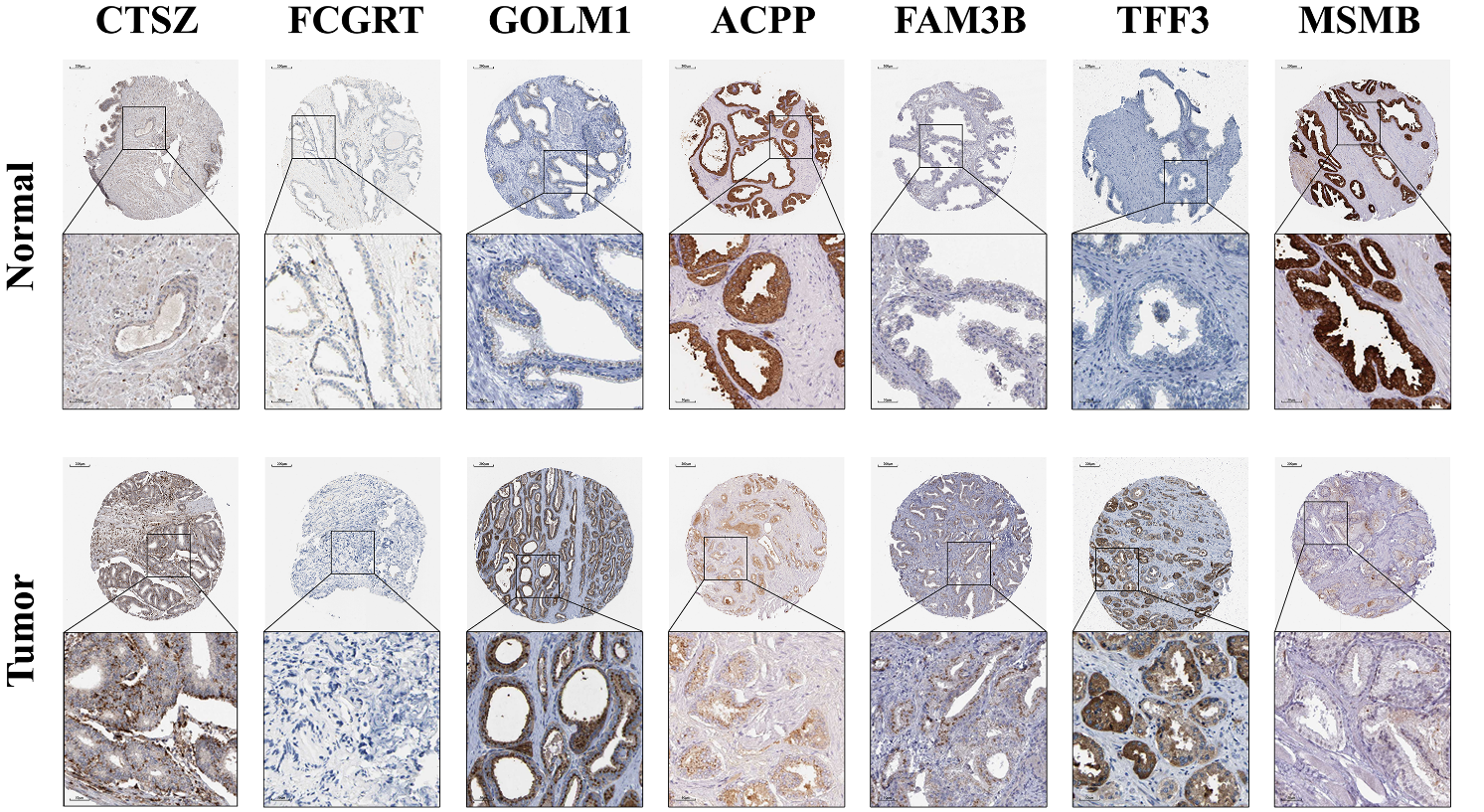


**Supplementary Figure 4. Immunohistochemistry based on the HPA database.** The protein levels of the nine MRMGPS-based genes based on the HPA database. HPA, The Human Protein Atlas.


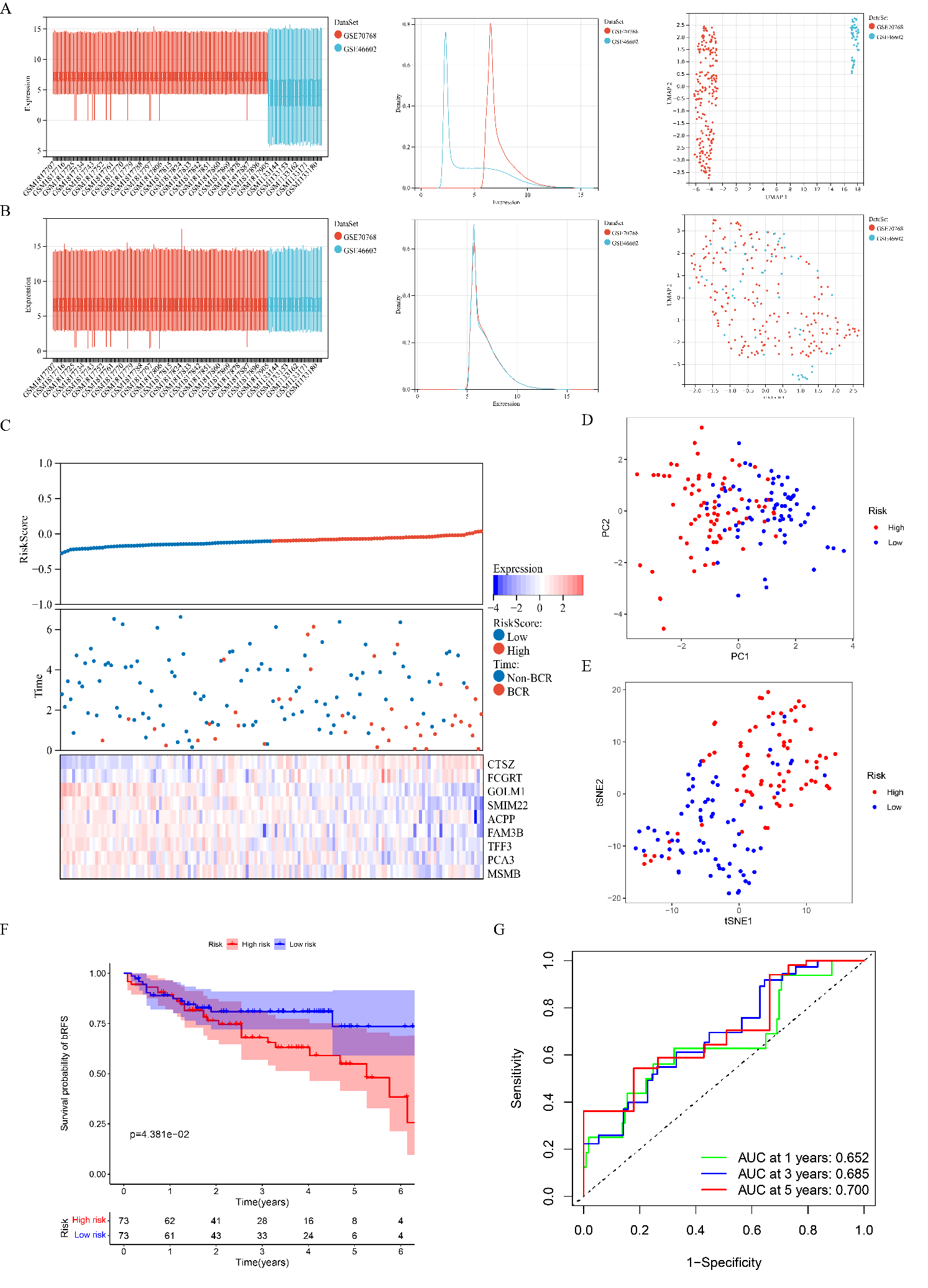


**Supplementary Figure 5. Validation of MRMGPS in GEO-Merged cohort.** **(A, B)** Comparison of two datasets (GSE70768 and GSE46602) before **(A)** and after **(B)** removing batch effect. **(C)** Relationship between risk score and follow-up time, BCR events, and changes of model genes expression in PCa patients. **(D)** PCA plot displaying the distribution of high- and low-risk groups. **(E)** t-SNE plot displaying the distribution of high- and low-risk groups. **(F)** Kaplan–Meier curves for the probability of bRFS grouped in high- and low-risk groups. **(G)** ROC curves for the predictive efficiency in 1-, 3-, and 5-year bRFS of MRMGPS. ROC, receiver operating characteristic; AUC, area under the ROC curve; BCR, biochemical recurrence; bRFS, biochemical recurrence-free survival.


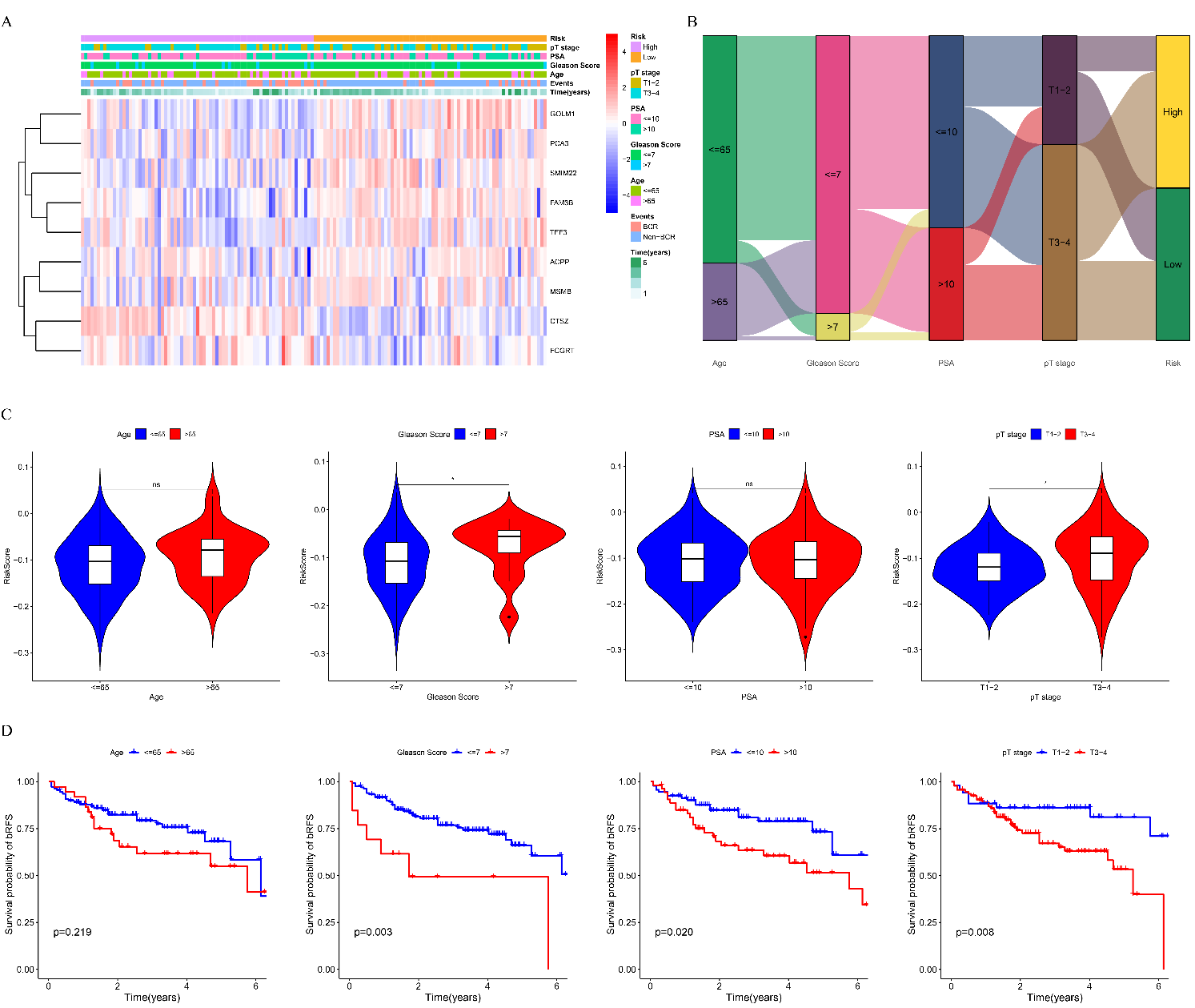


**Supplementary Figure 6. Correlation between MRMGPS and clinicopathological features in GEO-Merged cohort. (A)** The landscape of MRMGPS and clinicopathological features (pT stage, PSA, Gleason Score, Age). **(B)** Sankey diagram displaying for distribution of samples in different subgroups stratified by clinicopathological features. **(C)** Comparison of the risk score between different subgroups stratified by clinicopathological features. **(D)** Kaplan–Meier curves for the probability of bRFS stratified by clinicopathological features. PSA, prostate specific antigen. **P* <0.05, ** *P* < 0.01, *** *P* < 0.001, **** *P* < 0.0001. ns, not significant.


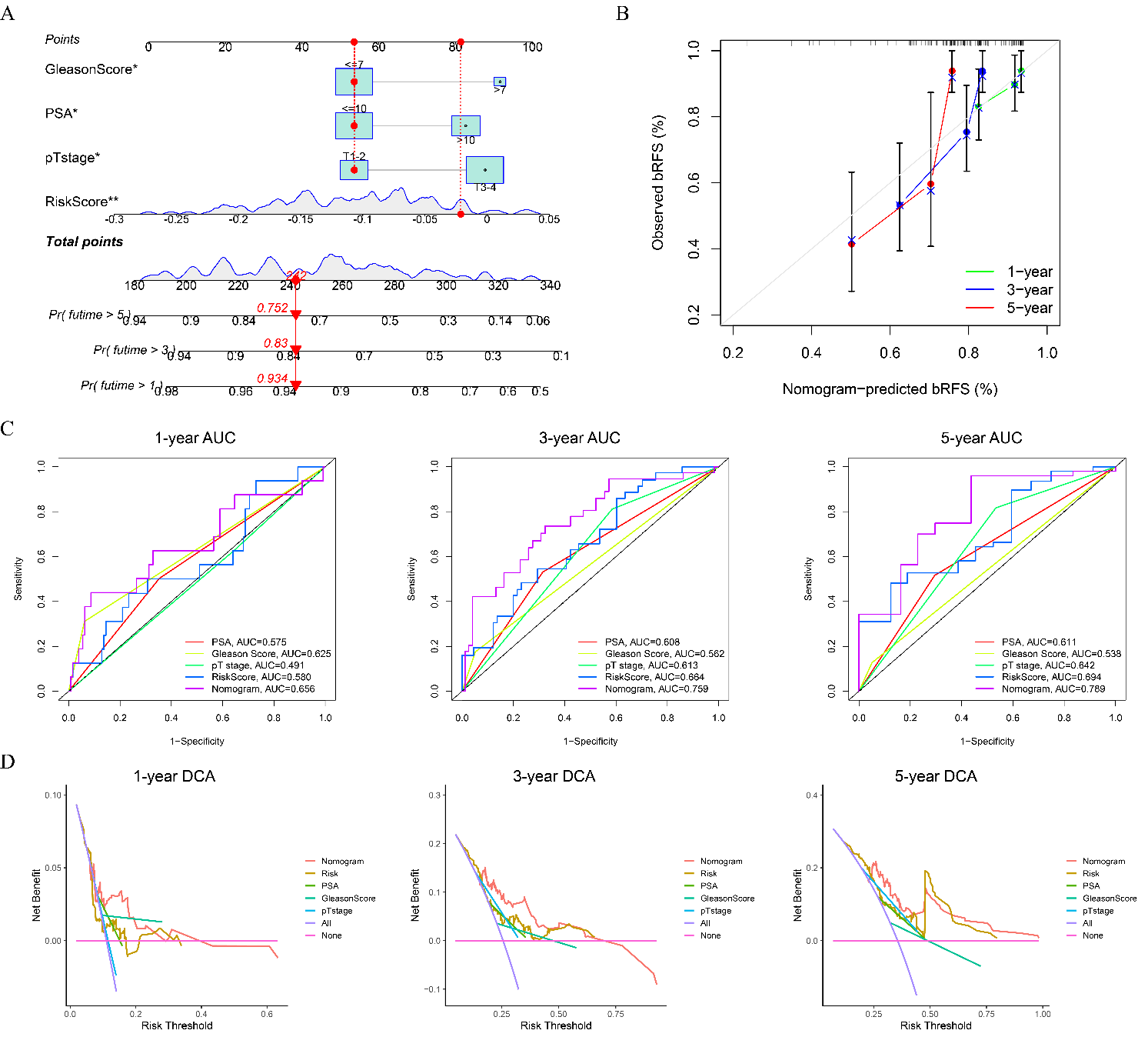


**Supplementary Figure 7. Construction of a nomogram in GEO-Merged cohort. (A)** The nomogram based on risk score, pT stage, PSA, and Gleason Score. **(B)** Calibration curves for the internal verification in 1-, 3-, and 5-year bRFS of the nomogram. **(C)** ROC curves for the predictive efficiency in 1-, 3-, and 5-year bRFS of the nomogram. **(D)** DCA curves for the net benefit in 1-, 3-, and 5-year bRFS of the nomogram. DCA, decision curve analysis; **P* <0.05, ** *P* < 0.01, *** *P* < 0.001, **** *P* < 0.0001. ns, not significant.

## Supplementary Tables

**Table S1.** The primers for RT-qPCR.

| **Genes** | **Forward Primer** | **Reverse Primer** |
| --- | --- | --- |
| **CTSZ** | 5′-CATCCCTGACGAGACCTG-3′ | 5′-GCATGTCCCACATTGGTTAAA-3′ |
| **FCGRT** | 5′‐AGCTCAAGTTCCGATTCCTG‐3′ | 5′‐GATCTGGCTGATGAATCTAGGTC‐3′ |
| **GOLM1** | 5′-CAGCGTGAAAAGCGGAATC-3′ | 5′-TCGGCCCTGTTGTGAAATA-3′ |
| **SMIM22** | 5′-ATGGCTGTGTCCACAGAGGA-3′ | 5′-GGGTTCCAGGGCCAAGTTAT-3′ |
| **ACPP** | 5′-TCGAAGCACAGACGTTGAC-3′ | 5′-AAGGCAGGTATAGCAACTGAT-3′ |
| **FAM3B** | 5′-CAAGATCTCCAAGGATTCG-3′ | 5′-TTTTTACAGATGCTTTCAG-3′ |
| **TFF3** | 5′-AACCGGGGCTGCTGCTTTGACTC-3′ | 5′-TCCTGCAGGGGCTTGAAACACCA-3′ |
| **PCA3** | 5′-ACACAGGAAGCACAAAAGG-3′ | 5′-GATGACCCAAGATGGCGGC-3′ |
| **MSMB** | 5′-AAATTTCATGTTGCACCCTTG-3′ | 5′-CCATTCACTGACAGAACAGGTC-3′ |
| **GADPH** | 5′-GACTCATGACCACAGTCCATGC-3′ | 5′-AGAGGCAGGGATGATGTTCTG-3′ |

**Table S2.** The gene sets with annotation of 29 immune cells and pathways.

| **Terms** | **Genes** |
| --- | --- |
| **aDCs** | CD83, LAMP3, CCL1 |
| **APC_co_inhibition** | C10orf54, CD274, LGALS9, PDCD1LG2, PVRL3 |
| **APC_co_stimulation** | CD40, CD58, CD70, ICOSLG, SLAMF1, TNFSF14, TNFSF15, TNFSF18, TNFSF4, TNFSF8, TNFSF9 |
| **B_cells** | BACH2, BANK1, BLK, BTLA, CD79A, CD79B, FCRL1, FCRL3, HVCN1, RALGPS2 |
| **CCR** | CCL16, TPO, TGFBR2, CXCL2, CCL14, TGFBR3, IL11RA, CCL11, IL4I1, IL33, CXCL12, CXCL10, BMPER, BMP8A, CXCL11, IL21R, IL17B, TNFRSF9, ILF2, CX3CR1, CCR8, TNFSF12, CSF3, TNFSF4, BMP3, CX3CL1, BMP5, CXCR2, TNFRSF10D, BMP2, CXCL14, CCL28, CXCL3, BMP6, CCL21, CXCL9, CCL23, IL6, TNFRSF18, IL17RD, IL17D, IL27, CCL7, IL1R1, CXCR4, CXCR2P1, TGFB1I1, IFNGR1, IL9R, IL1RAPL1, IL11, CSF1, IL20RA, IL25, TNFRSF4, IL18, ILF3, CCL20, TNFRSF12A, IL6ST, CXCL13, IL12B, TNFRSF8, IL6R, BMPR2, IFNE, IL1RAPL2, IL3RA, BMP4, CCL24, TNFSF13B, CCR4, IL2RA, IL32, TNFRSF10C, IL22RA1, BMPR1A, CXCR5, CXCR3, IFNA8, IL17REL, IFNB1, IFNAR1, TNFRSF1B, CCL17, IFNL1, IL16, IL1RL1, ILK, CCL25, ILDR2, CXCR1, IL36RN, IL34, TGFB1, IFNG, IL19, ILKAP, BMP2K, CCR10, ILDR1, EPO, CCR7, IL17C, IL23A, CCR5, IL7, EPOR, CCL13, IL2RG, IL31RA, TNFAIP6, IFNL2, BMP1, IL12RB1, TNFAIP8, IL4R, TNFRSF6B, TNFAIP8L1, TNFRSF10B, IFNL3, CCL5, CXCL6, CXCL1, CCR3, TNFSF11, CSF1R, IL21, IL1RAP, IL12RB2, CCL1, IL17RA, CCR1, IL1RN, TNFRSF11B, TNFRSF14, IL13, IL2RB, BMP8B, CCL2, IL24, IL18RAP, TGFBI, TNFSF10, TNFRSF11A, CXCL5, IL5RA, TNFSF9, IL1RL2, TNFRSF13C, IL36G, IL15RA, TNFRSF21, CXCL8, IL22RA2, TNFAIP8L2, IL18R1, IFNLR1, CXCR6, CCL3L3, TNFRSF1A, IL17RE, IFNGR2, IL17RC, TNFAIP8L3, ILVBL, TGFBRAP1, CCL4L1, CSF2RA, CCRN4L, CCL26, TNFAIP1, CCRL2, IFNA10, TNFRSF17, IFNA13, IL20, IL18BP, CCL3L1, TNFSF12-TNFSF13, IL5, IL23R, IL26, TNF, TGFA, CSF2, IL1F10, CXCL17, TNFSF13, IFNA4, IL37, IL12A, IL7R, IFNA1, IL1A, IL4, IL2, CCL22, CSF3R, IL10, IFNK, TGFB2, IL1R2, IL1B, IL17F, IL27RA, IL15, TNFSF8, IL36B, XCL1, CXCL16, TNFRSF19, IL3, CCL3, IFNA2, BMPR1B, IFNA21, TNFSF18, CCL8, IL17RB, TNFRSF25, IL22, IL10RB, IFNAR2, CCL18, IFNA16, CSF2RB, IL36A, TNFAIP3, IL13RA2, IL13RA1, CCR9, TNFRSF10A, IFNA7, IFNW1, XCL2, TNFSF14, CCR2, BMP15, BMP10, CCL15-CCL14, TGFBR1, IFNA5, BMP7, IFNA14, IL20RB, IL10RA, IFNA17, CCR6, TGFB3, CCL15, CCL4, CCL27, TNFRSF13B, TNFAIP2, IL31, IL17A, TNFSF15, CCL19, IFNA6, IL9 |
| **CD8+_T_cells** | CD8A |
| **Check-point** | IDO1, LAG3, CTLA4, TNFRSF9, ICOS, CD80, PDCD1LG2, TIGIT, CD70, TNFSF9, ICOSLG, KIR3DL1, CD86, PDCD1, LAIR1, TNFRSF8, TNFSF15, TNFRSF14, IDO2, CD276, CD40, TNFRSF4, TNFSF14, HHLA2, CD244, CD274, HAVCR2, CD27, BTLA, LGALS9, TMIGD2, CD28, CD48, TNFRSF25, CD40LG, ADORA2A, VTCN1, CD160, CD44, TNFSF18, TNFRSF18, BTNL2, C10orf54, CD200R1, TNFSF4, CD200, NRP1 |
| **Cytolytic_activity** | PRF1, GZMA |
| **DCs** | CCL17, CCL22, CD209, CCL13 |
| **HLA** | HLA-E, HLA-DPB2, HLA-C, HLA-J, HLA-DQB1, HLA-DQB2, HLA-DQA2, HLA-DQA1, HLA-A, HLA-DMA, HLA-DOB, HLA-DRB1, HLA-H, HLA-B, HLA-DRB5, HLA-DOA, HLA-DPB1, HLA-DRA, HLA-DRB6, HLA-L, HLA-F, HLA-G, HLA-DMB, HLA-DPA1 |
| **iDCs** | CD1A, CD1E |
| **Inflammation-promoting** | CCL5, CD19, CD8B, CXCL10, CXCL13, CXCL9, GNLY, GZMB, IFNG, IL12A, IL12B, IRF1, PRF1, STAT1, TBX21 |
| **Macrophages** | C11orf45, CD68, CLEC5A, CYBB, FUCA1, GPNMB, HS3ST2, LGMN, MMP9, TM4SF19 |
| **Mast_cells** | CMA1, MS4A2, TPSAB1 |
| **MHC_class_I** | B2M, HLA-A, TAP1 |
| **Neutrophils** | EVI2B, HSD17B11, KDM6B, MEGF9, MNDA, NLRP12, PADI4, SELL, TRANK1, VNN3 |
| **NK_cells** | KLRC1, KLRF1 |
| **Parainflammation** | CXCL10, PLAT, CCND1, LGMN, PLAUR, AIM2, MMP7, ICAM1, MX2, CXCL9, ANXA1, TLR2, PLA2G2D, ITGA2, MX1, HMOX1, CD276, TIRAP, IL33, PTGES, TNFRSF12A, SCARB1, CD14, BLNK, IFIT3, RETNLB, IFIT2, ISG15, OAS2, REL, OAS3, CD44, PPARG, BST2, OAS1, NOX1, PLA2G2A, IFIT1, IFITM3, IL1RN |
| **pDCs** | CLEC4C, CXCR3, GZMB, IL3RA, IRF7, IRF8, LILRA4, PHEX, PLD4, PTCRA |
| **T_cell_co-inhibition** | BTLA, C10orf54, CD160, CD244, CD274, CTLA4, HAVCR2, LAG3, LAIR1, TIGIT |
| **T_cell_co-stimulation** | CD2, CD226, CD27, CD28, CD40LG, ICOS, SLAMF1, TNFRSF18, TNFRSF25, TNFRSF4, TNFRSF8, TNFRSF9, TNFSF14 |
| **T_helper_cells** | CD4 |
| **Tfh** | PDCD1, CXCL13, CXCR5 |
| **Th1_cells** | IFNG, TBX21, CTLA4, STAT4, CD38, IL12RB2, LTA, CSF2 |
| **Th2_cells** | PMCH, LAIR2, SMAD2, CXCR6, GATA3, IL26 |
| **TIL** | ITM2C, CD38, THEMIS2, GLYR1, ICOS, F5, TIGIT, KLRD1, IRF4, PRKCQ, FCRL5, SIRPG, LPXN, IL2RG, CCL5, LCK, TRAF3IP3, CD86, MAL, LILRB1, DOK2, CD6, PAG1, LAX1, PLEK, PIK3CD, SLAMF1, XCL1, GPR171, XCL2, TBX21, CD2, CD53, KLHL6, SLAMF6, CD40, SIT1, TNFRSF4, CD79A, CD247, LCP2, CD3D, CD27, SH2D1A, FYB, ARHGAP30, ACAP1, CST7, CD3G, IL2RB, CD3E, FCRL3, CORO1A, ITK, TCL1A, CYBB, CSF2RB, IKZF1, NCF4, DOCK2, CCR2, PTPRC, PLAC8, NCKAP1L, IL7R, 6-Sep, CD28, STAT4, CD8A, LY9, CD48, HCST, PTPRCAP, SASH3, ARHGAP25, LAT, TRAT1, IL10RA, PAX5, CCR7, DOCK11, PARVG, SPNS1, CD52, HCLS1, ARHGAP9, GIMAP6, PRKCB, MS4A1, GPR18, TBC1D10C, GVINP1, P2RY8, EVI2B, VAMP5, KLRK1, SELL, MPEG1, MS4A6A, ARHGAP15, MFNG, GZMK, SELPLG, TARP, GIMAP7, FAM65B, INPP5D, ITGA4, MZB1, GPSM3, STK10, CLEC2D, IL16, NLRC3, GIMAP5, GIMAP4, IFFO1, CFH, PVRIG, CFHR1 |
| **Treg** | IL12RB2, TMPRSS6, CTSC, LAPTM4B, TFRC, RNF145, NETO2, ADAT2, CHST2, CTLA4, NFE2L3, LIMA1, IL1R2, ICOS, HSDL2, HTATIP2, FKBP1A, TIGIT, CCR8, LTA, SLC35F2, IL21R, AHCYL1, SOCS2, ETV7, BCL2L1, RRAGB, ACSL4, CHRNA6, BATF, LAX1, ADPRH, TNFRSF4, ANKRD10, CD274, CASP1, LY75, NPTN, SSTR3, GRSF1, CSF2RB, TMEM184C, NDFIP2, ZBTB38, ERI1, TRAF3, NAB1, HS3ST3B1, LAYN, JAK1, VDR, LEPROT, GCNT1, PTPRJ, IKZF2, CSF1, ENTPD1, TNFRSF18, METTL7A, KSR1, SSH1, CADM1, IL1R1, ACP5, CHST7, THADA, CD177, NFAT5, ZNF282, MAGEH1 |
| **Type_I_IFN_Reponse** | DDX4, IFIT1, IFIT2, IFIT3, IRF7, ISG20, MX1, MX2, RSAD2, TNFSF10 |
| **Type_II_IFN_Reponse** | GPR146, SELP, AHR |

**Table S3.** The RNA-modified genes.

| **Type** | **Genes** |
| --- | --- |
| **m6A_writer** | METTL3, RBM15B, ZC3H13, METTL14, CBLL1, RBM15, WTAP |
| **m6A_eraser** | ALKBH5, FTO |
| **m6A_reader** | HNRNPC, YTHDC1, HNRNPA2B1, YTHDF1, YTHDC2, FMR1, ELAVL1, YTHDF2, YTHDF3, IGF2BP1, LRPPRC |
| **m1A_writer** | TRMT10C, TRMT61B, TRMT6, TRMT61A |
| **m1A_reader** | YTHDF1, YTHDF2, YTHDF3, YTHDC1 |
| **m1A_eraser** | ALKBH1, ALKBH3 |
| **m5C_writer** | NOP2, NSUN2, NSUN3, NSUN4, NSUN5, NSUN6, NSUN7, DNMT1, TRDMT1, DNMT3A, DNMT3B |
| **m5C_eraser** | TET2 |
| **m5C_reader** | ALYREF |

**Table S4.** The main clinicopathological features of PCa patients in the study.

| **Variables** | **TCGA** | **GSE70768** | **GSE46602** |
| --- | --- | --- | --- |
| **Total** | 346 | 110 | 36 |
| **Age (years)** |  |  |  |
| ≤65 | 247 | 86 | 23 |
| >65 | 99 | 24 | 13 |
| **Gleason Score** |  |  |  |
| ≤7 | 190 | 101 | 32 |
| >7 | 156 | 9 | 4 |
| **PSA (ng/mL)** |  |  |  |
| ≤10 | 335 | 83 | 9 |
| >10 | 11 | 27 | 27 |
| **Status** |  |  |  |
| BCR | 37 | 19 | 22 |
| Non-BCR | 309 | 91 | 14 |
| **Pathological T stage** |  |  |  |
| T1-2 | 121 | 33 | 19 |
| T3-4 | 225 | 77 | 17 |
| **Pathological N stage** |  |  |  |
| N0 | 287 | - | - |
| N1 | 59 | - | - |

BCR, biochemical recurrence; PSA, prostate specific antigen.

**Table S5.** The 21 clusters with annotation in scRNA-seq.

| **Cluster-IDs** | **Cluster-Labels** | **Marker Genes** |
| --- | --- | --- |
| 0, 1, 3, 4, 5, 6, 8, 9, 13, 15, 17, 20 | Epithelial_cells | KLK3, KLK2, AZGP1, SPON2, TSPAN1, PDLIM5, ACPP, KRT18, TFF3, TRPM4, FXYD3, KLK4, GDF15, NKX3-1, MSMB, TRGC1, RDH11, KRT8, TMPRSS2, STEAP2, DCXR, IGF1, AGR2, MESP1, PCA3, MPC2, LRRC26, SORD, CLDN3, ELF3, H2AFJ, CLDN4, FOLH1, KLK11, TACSTD2, MGST1, NUDT8, SLC45A3, SPDEF, SMIM22, HSD17B6, SNHG19, SMS, NCAPD3, GOLM1, HEBP2, NUPR1, ABHD2, FASN, ALDH1A3, DHRS7, PRAC1, SLC39A6, PMEPA1, PART1, OR51E2, ABCC4, TRPM8, SPINT2, P4HB, FAM3B, ZG16B, PPP3CA, NEDD4L, SLC4A4, SCD, DHCR24, EPCAM, FOXA1, C19orf48, MIPEP, LINC01088, KLK12, KRT19, MCCC2, CXADR, TSPAN8, MAOA, TMEM141, ALOX15B, SPOCK1, TPD52, SNRPN, DBI, ERGIC1, SNHG25, MLPH, UQCRQ, TSTD1, STEAP1, BNIP3, TRIB1, XBP1, CRISP3, PCBD1, STRA13, RPL7A, TM7SF2, PERP, CD24, AR, CREB3L4, TMEFF2, HOXB13, RPLP0, DDT, RP11-12G12.7, ARG2, HIST1H1C, RAMP1, NME4, PPDPF, TSPAN13, PTPRF, ARHGEF26, TCEA3, PCAT4, TBX3, MARCKSL1, ARFGEF3, ERBB3, HPN, SLC44A4, ENTPD5, RPL8, MDK, CKB, C1QTNF9B-AS1, SEC11C, IGFBP2, BMPR1B, TMSB15A, SNHG8, HIST3H2A, ADI1, DSP, ACAD8, SEMA3C, CCNG2, DUSP1, NR3C1, GYPC, FLNA, CELF2, CKLF, LGALS3, HLA-F, LINC00152, PHLDA1, GNAI2, PSMB9, ACTB, STK4, ZEB2, CLEC2B, HIST1H4C, ARPC2, ISG15, CD48, PNRC1, COTL1, 7-Sep, TAGLN2, AKAP13, MYL12A, CALM1, TMSB10, S100A10, TACC1, CD53, TGFBR2, PTRF, CD44, MSN, BIRC3, TUBA1A, CTSC, ZFP36, DDIT4, ARPC1B, HLA-DMA, GIMAP7, SGK1, SAMSN1, STOM, VAMP5, BST2, ELF1, EPAS1, CRIP2, MEF2C, NR4A2, JUNB, LDHB, IFI16, HCST, ITM2B, GSTP1, TCF4, TNFAIP3, GMFG, EMP3, ITM2A, DUSP2, CD37, HLA-DQB1, PTPRC, ID3, IFITM1, KLF2, HLA-DRB5, SOCS3, LAPTM5, SH3BGRL3, IGFBP4, FXYD5, CREM, CAV1, TMSB4X, ID2, ZFP36L2, GNG11, CRIP1, SLC2A3, ANXA1, BTG1, HLA-C, TIMP1, IL32, TXNIP, TSC22D3, MT2A, CD52, IFITM3, ARHGDIB, IFITM2, GSN, CYBA, TIMP3, A2M, TYROBP, S100A6, SPRY1, HLA-B, HLA-DPA1, HLA-E, B2M, CD69, RGCC, MGP, HLA-DPB1, HLA-A, LGALS1, SPARC, S100A4, IFI27, HLA-DRB1, RGS1, CXCR4, SRGN, HLA-DRA, SPARCL1, VIM, CD74, IGFBP7, ZNF331, THYN1, FTH1, TPM3, DDX24, NFKBIA, PLA2G2A, FCGRT, APOE, ID1, GADD45B, ATP1B3, LMNA, MYADM, CNN3, RGS2, REL, ITGB1, HSP90AA1, CLU, IER3, CTSB, DNAJB1, MT1E, MT1X, HSPH1, CALD1, HSPA6, CTSD, FTL |
| 2 | T_cells | CCL5, CXCR4, IL32, NKG7, IL7R, CCL4, CD3D, CD52, ZFP36L2, TRAC, TRBC1, RGS1, PTPRC, TRBC2, KLRB1, CREM, DUSP2, CD2, GZMA, BTG1, HCST, CD3E, TSC22D3, S100A4, SRGN, CST7, HLA-A, HSP90AA1, DUSP4, GZMK, GZMH, CD7, TNFAIP3, CORO1A, CYTIP, DNAJB1, FYB, B2M, CD69, LTB, HSPH1, CD3G, ZNF331, RUNX3, LEPROTL1, HLA-B, HLA-C, CRIP1, ARHGDIB, ISG20, FYN, DOK2, RARRES3, IL2RG, CD53, STK4, ANXA1, CLEC2D, PPP2R5C, CYBA, ELF1, ID2, GPR171, TUBA4A, CD48, ACAP1, TXNIP, SH3BGRL3, RP11-138A9.2, CELF2, RAC2, CTSW, EVL, STK17A, SPOCK2, ARL4C, SYTL3, SLA, CD44, GMFG, BIRC3, SAMSN1, PIK3IP1, LSP1, RORA, AC016831.7, CD96, STK17B, AKNA, LCK, DDX24, CLEC2B, RP11-138A9.1, ETS1, HLA-F, CD37, STAT4, HLA-E, GZMM, TMSB4X, GLIPR1, CD247, GPR183, WIPF1, RHOH, SARAF, PSMB9, PTPN7, LAPTM5, GPSM3, ADGRE5, EVI2A, IKZF3, ALOX5AP, PRDM1, FXYD5, UCP2, IKZF1, LDHB, CARD16, EMP3, CALM1, ITM2A, LINC00152, RGCC, ITGB2, IFITM1, EVI2B, HDLBP, ACSL3, CREB3L4, TMSB15A, PTPRF, GRN, HSBP1, RPL8, RPLP0, FLNB, FKBP2, TM9SF3, KDELR2, HOXB13, STRA13, BNIP3, RAC1, DDT, MGST2, RPL12, GATA2, RPL37A, BASP1, STEAP1, TBX3, CCNG2, ADI1, CXADR, TNFSF10, RPL23, WBP5, H1F0, RPL7A, CKB, HES4, C19orf48, AR, SMIM14, YWHAE, CANX, ERGIC1, CCND1, PCBD1, CD151, ALOX15B, DHCR24, EPCAM, SLC9A3R2, ASAH1, HSP90B1, SYNGR2, CYB5A, RAB13, NME4, SLC4A4, FOXA1, INSR, TPM1, CD63, MAOA, MLPH, NPC2, ZG16B, FAM3B, MARCKS, TMEM141, PSAP, FTL, RAMP1, PRSS23, MARCKSL1, MCCC2, PEBP1, MDK, GPX1, TSPAN13, UQCRQ, CYR61, SLC39A6, SPOCK1, DBI, P4HB, SCD, FASN, SMIM22, TRIB1, NFIB, CPE, KLF4, NEDD4L, OR51E2, BCAM, HSD17B6, RHOB, HEBP2, PPP3CA, ALDH1A3, CLDN4, ABHD2, PART1, BRI3, TRPM8, ELF3, CLDN3, DSTN, APP, NGFRAP1, NUDT8, APLP2, MIPEP, SPDEF, EGR1, NEAT1, PMEPA1, ABCC4, SEPP1, SPINT2, DCXR, LRRC26, SNHG19, NPDC1, TSC22D1, SORD, TM4SF1, TACSTD2, PLPP1, GOLM1, SMS, PTMS, LINC01088, HES1, SOX4, SLC45A3, IGFBP2, KLK4, FOLH1, MGST1, MPC2, MESP1, AGR2, TMPRSS2, NKX3-1, CALD1, KRT8, STEAP2, TRGC1, PCA3, CD9, TRPM4, RDH11, GDF15, ACPP, FXYD3, H2AFJ, KRT18, NUPR1, AZGP1, CST3, TSPAN1, TFF3, PDLIM5, KLK2, KLK3, HSPA8, MAFB, IGF1, PTGER4, BANK1, NR3C1, TSPAN8, CEBPD, ATF3, HMGB2, AKAP13, MT2A, MT1X, DHRS7, SPON2, KRT19, SRSF7, LIMD2, HSPA1A, PIK3R1, AREG, MSMB, DOCK8, KIAA1551, SOCS1, CD24, HSPE1, CDC42SE2, CACYBP, SMCHD1, CCND3, PRAC1, DDIT4, SYNE2, IFITM3, YPEL5, CCNH, TSPYL2, PIP4K2A, IDS, CNOT6L, FNBP1, SMAP2, HSPD1, DNAJB6, ARPC2, DNAJA1, RNF213, PPP1R2, REL, HIST1H4C, RNF19A, RGS2, EML4, COTL1, ADIRF, CLU, SLC38A1, JUNB, SP100, PDE4B, MT1E, NR4A2, CKLF, CXCL8, NCAPD3, MT1F, EMB, FABP5, PLA2G2A, ZC3HAV1, FAM177A1, BCAS2, ATP1B3, TMEM2, HSPA6, CHORDC1, GPBP1, HSPA1B, PMAIP1, HLA-DRA, MT1G |
| 7, 14, 19 | Endothelial_cells | IFI27, SPRY1, SPARCL1, PLVAP, RAMP2, GNG11, ACKR1, VWF, IGFBP7, MGP, RGCC, GSN, IFITM3, A2M, ENG, IGFBP4, FLT1, AQP1, CLDN5, RNASE1, HSPG2, IGFBP3, EMCN, SPARC, HLA-E, PLPP3, SOCS3, PECAM1, SDPR, ADGRL4, TCF4, CTGF, RAMP3, COL4A1, VIM, EPAS1, INSR, CAV1, CRIP2, CLEC14A, IFITM2, TIMP3, ID3, SLC9A3R2, EGFL7, KLF2, VAMP5, ID1, TM4SF1, TGFBR2, GIMAP7, EMP1, SLC2A3, ECSCR.1, ENPP2, IFITM1, CCL14, ITM2A, CLU, ESAM, CD93, PTPRB, SLCO2A1, LDB2, STOM, HYAL2, COL4A2, TSPAN7, CD34, FKBP1A, PODXL, HLA-DRB5, CNN3, HLA-B, CALCRL, ITM2B, SEC14L1, PTRF, RBP7, SPTBN1, PCAT19, LIFR, ANGPT2, COL15A1, STC1, TMEM204, TMEM88, APP, CD74, TM4SF18, THBD, VWA1, MEF2C, RDX, C10orf10, BST2, TXNIP, PLAT, TACC1, CD59, EDNRB, HLA-DRB1, PLPP1, HLA-A, GIMAP4, NOSTRIN, NOTCH4, PDLIM1, B2M, CXorf36, ADGRF5, EDN1, ARHGAP29, NNMT, MTUS1, APOLD1, HLA-C, PALMD, ETS2, IFI16, KDR, IFI6, MMRN2, IL6ST, S100A16, CDH5, GJA1, TSC22D1, SH3BP5, EHD4, ICAM2, CYYR1, MYL12A, XAF1, NCOA7, ANXA2, JAM2, LMCD1, ITGA6, PDK4, TSHZ2, GNAI2, HEG1, ZFP36, TMSB10, S100A13, PDGFD, ISG15, WWTR1, FAM167B, UACA, S100A10, S1PR1, PRCP, GAS6, COX7A1, ITGB1, SNCG, KCTD12, MSN, S100A6, SRP14, NEDD9, MGST2, CCDC85B, LIMS2, TGFBR3, CALM1, THY1, HTRA1, ELK3, FCGRT, PTPRG, TFPI, NRP1, CTSC, YBX3, LRRC32, SOX17, FAM107A, SWAP70, TAGLN2, ABCG2, ROBO4, DLC1, ADCY4, PCDH17, MYH9, MYL12B, NFIB, ARL4A, TIMP1, PLXND1, FAM198B, CAV2, ASAP1, LEPROT, CDC37, BMPR2, TIE1, RND1, TGM2, MYCT1, ENTPD1, LMO2, NUAK1, MEIS2, EMP2, IL33, GRB10, DOCK9, TIMP2, ITGA5, NES, PRSS23, CDA, PRKCDBP, SOX18, PIK3R3, PPFIBP1, ERG, AKR1C3, DPYSL2, NRP2, GIMAP1, LUZP1, MCAM, CLIC4, IFI44L, KIAA0355, GBP4, NASP, TMEM255B, ETS1, ARPC1B, BCAM, RBMS1, ZFP36L1, IL3RA, ZEB1, BHLHE40, AP1S2, KCNN3, DUSP6, GSTP1, CX3CL1, FXYD5, PSMB9, TNS2, TINAGL1, UPP1, DLL4, MKL2, JUNB, ID2, PERP, RPSA, HIST3H2A, RPL7A, TMSB15A, ARHGEF26, EZR, ERGIC1, HOXB13, PEBP1, BASP1, VAMP8, RPLP0, CKB, STEAP1, HIST1H1C, PCBD1, PPDPF, P4HB, SERP1, RGS10, NEDD4L, CXADR, MCCC2, ALOX15B, EPCAM, TPD52, XBP1, DHCR24, SPOCK1, HEBP2, ZG16B, C19orf48, MLPH, FOXA1, ALDH1A3, SLC4A4, RAMP1, PMEPA1, FASN, ABCC4, SNHG25, GOLM1, SCD, NUDT8, OR51E2, DHRS7, SMIM22, DBI, TRPM8, SNHG19, PART1, FTL, SLC39A6, MIPEP, SPDEF, NUPR1, TACSTD2, TSTD1, CLDN3, FOLH1, ELF3, LRRC26, SMS, SPINT2, SORD, SLC45A3, CLDN4, MESP1, MGST1, MPC2, TMPRSS2, KLK4, RDH11, STEAP2, PCA3, KRT8, NKX3-1, AGR2, DCXR, TRPM4, GDF15, ACPP, H2AFJ, KRT18, PDLIM5, FXYD3, TSPAN1, TRGC1, TFF3, AZGP1, SPON2, MSMB, KLK2, KLK3, FN1, HES1, HSD17B6, SNRPN, TMEM141, BNIP3, CEBPD, FAM3B, FTH1, PPP3CA, ARGLU1, ABHD2, IGF1, TSPAN8, HSPE1, CD24, KRT19, LINC01088, ABL2, CD44, ADAMTS1, PRAC1, DDIT4, CDKN1A, DUSP23, EFNB2, IVNS1ABP, MT1F, AKAP12, RGS2, MT1G, HSPA6, CXCL8, CXCL2, PLA2G2A |
| 10 | Macrophage | APOC1, APOE, CCL3, HLA-DRA, C1QB, HLA-DPB1, TYROBP, C1QA, HLA-DPA1, CD74, G0S2, IL1B, CCL3L3, LYZ, FTL, HLA-DRB1, C1QC, AIF1, SPP1, CTSB, CXCL8, FCER1G, HLA-DQA1, GPNMB, MS4A6A, CTSD, HLA-DQB1, CCL4L2, GPX1, CD68, MS4A7, LST1, CTSS, NPC2, CXCL2, CXCL3, HLA-DMA, LGALS1, PSAP, CCL4, CYBA, PLAUR, IER3, BCL2A1, HLA-DRB5, HLA-DMB, SOD2, S100A9, LAPTM5, GPR183, CTSZ, CD14, LGMN, IGSF6, CSTB, FCGR2A, GRN, FTH1, LGALS3, LIPA, CAPG, CD83, MS4A4A, C15orf48, RNASE6, CST3, MAFB, FCGR3A, HLA-DQA2, COTL1, SRGN, TREM2, CTSL, SLC40A1, SGK1, PLIN2, TMSB4X, ITGB2, RGS1, PHACTR1, C5AR1, CFD, OLR1, PLEK, FAM26F, DAB2, RNASET2, FCGRT, HMOX1, TMEM176B, TYMP, ACP5, GPR34, IFI30, LY96, CYBB, RGS2, FGL2, ALOX5AP, C1orf162, GLUL, LY86, MSR1, S100A11, SPI1, PYCARD, ARPC1B, AKR1B1, FOLR2, IER5, S100A4, FYB, TMSB10, TNFAIP2, MARCKS, LSP1, CXCL16, RNF130, CTSH, SDS, CTSC, GSTP1, RAB31, KCTD12, SAT1, CPVL, SH3BGRL3, LILRB4, LITAF, VSIG4, TGFBI, UCP2, ACTB, TNF, CD163, CSF1R, EMP3, VAMP8, HCLS1, AP1S2, VIM, GLIPR1, OGFRL1, IL18, MNDA, OTOA, LAIR1, ITGAX, ADAP2, CD4, PLTP, RASSF4, FAM49B, TMEM176A, CECR1, CD86, CLEC7A, C3AR1, SERPINA1, CREG1, CSF2RA, GMFG, TNFSF13B, ZEB2, TPP1, FXYD5, MPEG1, SERPINB9, PTPRE, CPM, FCGR2B, SLC1A3, SAMHD1, CCRL2, CD53, LGALS9, AXL, CKLF, ARPC3, CD84, B2M, MGAT1, GLRX, LCP2, ARRB2, MFSD1, CD37, ARL4C, C3, SNX10, NPDC1, DSTN, KRT18, TSPAN1, PDLIM5, KLK2, ARPC2, NINJ1, KLK3, LIMS1, ITM2B, LINC00936, RGS10, ID2, SDCBP, CEBPB, C12orf57, YWHAH, KRT8, TRPM4, PLPP1, PPDPF, TMPRSS2, BCAM, NFIB, PMEPA1, NKX3-1, IGFBP2, SPDEF, REL, TRGC1, ATP6V1F, FXYD3, TNFAIP3, SORD, AZGP1, STEAP2, ALDH1A3, SLC45A3, MESP1, NFKBIA, CLDN3, ASAH1, MARCKSL1, AREG, TSPAN13, TACSTD2, MAOA, PRDX2, SLC39A6, H2AFJ, PERP, PART1, LRRC26, KLK4, HEBP2, SLC9A3R2, FASN, GOLM1, PLD3, CALD1, SNHG19, MPC2, ZG16B, RDH11, SMIM22, ELF3, TSTD1, TPD52, CLDN4, DCXR, MDK, ACPP, PRSS23, TRPM8, C19orf48, TNFSF10, NUDT8, SNRPN, FOLH1, TPM1, MGST1, ABCC4, PPP3CA, HSPA6, SPON2, FAM3B, SPOCK1, MCCC2, OR51E2, NEDD4L, TFF3, CPE, SNHG8, TSC22D1, GDF15, CYR61, RAMP1, DHRS7, PCA3, HSD17B6, TM4SF1, LINC01088, MIPEP, APP, KRT19, AGR2, CLU, ABHD2, PRAC1, ADIRF, HSPA1B, MSMB, MT1G, IGF1, PLA2G2A, THYN1 |
| 11 | Tissue_stem_cells | RGS5, TAGLN, ACTA2, MYL9, TPM2, CALD1, MYH11, IGFBP7, NDUFA4L2, C11orf96, TIMP1, HIGD1B, COL1A1, COL1A2, MFGE8, ADIRF, COL3A1, TIMP3, BGN, PDGFRB, DCN, LGALS1, SPARC, CRISPLD2, NOTCH3, SPARCL1, SOD3, PLN, PPP1R14A, MYLK, TPM1, COX4I2, CRIP1, MGP, FRZB, LHFP, CPM, CAV1, MAP1B, COL6A2, PLAC9, IGFBP5, GJA4, FLNA, MCAM, IFITM3, COL4A2, IGFBP6, PHLDA1, MEG3, COL18A1, TPPP3, CSRP2, VIM, RERGL, WFDC1, PRKCDBP, 7-Sep, COL4A1, PTRF, FILIP1L, SERPING1, TNS1, HOPX, SYNPO2, MT1M, CEBPD, FXYD1, SELM, A2M, AC013461.1, MT1A, PGF, EPS8, RARRES2, RGS16, TINAGL1, COL6A1, 11-Sep, ISYNA1, AEBP1, PCOLCE, ADAMTS1, COX7A1, THY1, CRYAB, ADAMTS4, FHL1, GSN, ITGB1, KANK2, S100A6, CARMN, RCAN2, NEXN, GEM, ID3, LMOD1, MEF2C, COL6A3, EFHD1, CCDC80, TUBA1A, GJC1, PTN, CRIP2, EHD2, GSTP1, CPE, GUCY1A2, MYL6, NDRG2, C1QTNF1, EBF1, C1R, GPX3, C1S, CNN3, FILIP1, IGFBP4, SLIT3, CNN1, PALLD, CAMK2N1, NTRK2, ID4, IFITM2, CLMN, KIAA0040, FN1, EPAS1, ITGA7, TGFB1I1, LTBP1, LGI4, MSRB3, COL14A1, GAS6, RBPMS, C2orf40, PRRX1, CAV2, NID1, STOM, FAM162B, MRVI1, LDHB, RND3, 4-Sep, FXYD6, PKIG, TBX2, AXL, PLXDC1, ADCY3, PDLIM1, ARHGEF17, PDLIM7, ITGA1, TBX2-AS1, CDH6, PMP22, DKK3, RRAD, NREP, C9orf3, RERG, ARHGAP10, NFASC, MGLL, ANXA6, EDNRA, GPR20, PDE5A, RPL36, RPL28, RPS21, RPS18, RPS29, SERP1, TSTD1, SPINT2, TMPRSS2, NKX3-1, KRT8, TRPM4, KRT18, PDLIM5, FXYD3, TSPAN1, TRGC1, AZGP1, TFF3, KLK2, KLK3, RHOB, PDK4, MGST1, TACSTD2, APOLD1, MPC2, SPDEF, STEAP2, TSPAN13, SLC45A3, MESP1, RPL12, NR2F2, SORD, GADD45B, PDLIM3, LGALS3BP, TNFSF10, MLPH, DCXR, PPP3CA, LRRC26, TESC, JUNB, CLDN3, PTP4A3, PPP1R12A, KLK4, FOXA1, TIMP2, VAMP8, LAPTM4A, LMNA, VCL, AKAP12, TACC1, EID1, PART1, ARID5B, CXADR, EPCAM, DSTN, NEDD4L, ABCC4, FLNB, PCA3, RDH11, SCD, CLDN4, MGST3, FKBP5, SLC4A4, SH3BGRL, TPD52, SLC39A6, TSC22D1, CEBPB, MYO1B, ERGIC1, GOLM1, PPP1R12B, S100A4, DHCR24, SMIM22, FOLH1, C19orf48, SAT1, TRPM8, ACTN1, ZG16B, ELF3, FASN, TM7SF2, MCCC2, NUDT8, GDF15, TLN1, AGR2, SNHG8, SNHG25, HIST1H4C, NPDC1, ALDH1A3, STEAP1, ALOX15B, MSMB, SOCS3, DBI, SMS, MAOA, PERP, LBH, ZBTB16, DHRS7, PTEN, JADE1, HEBP2, NR4A1, OR51E2, H2AFJ, ACPP, KLF9, FOS, MIPEP, TMEM141, SPOCK1, OAZ2, SPON2, HCFC1R1, MTHFD2, CYB5R3, UTRN, FAM3B, TPM4, P4HB, HSD17B6, S100A10, SNHG19, KRT19, ZFAND5, CRTAP, PLPP1, YBX3, CDKN1A, KLHL23, IGF1, JUN, PRAC1, EGR1, MYLIP, PHLDA2, LINC01088, CSRP1, CD74, FABP5, HLA-DRA, SBDS, SERTAD1, BTG2, CXCL8, SORBS2, HLA-DPB1, MYC, SRGN, ZNF331, MT1G |
| 12 | CMP | TPSB2, TPSAB1, CPA3, CD69, MS4A2, KIT, RGS13, ANXA1, HPGDS, SLC18A2, FCER1G, RGS1, GATA2, SRGN, S100A4, GPR65, VWA5A, ALOX5AP, SAMSN1, ANKRD28, NFKBIZ, LMO4, SGK1, C1orf186, LTC4S, CD44, TYROBP, RP11-354E11.2, ACSL4, IL1RL1, FXYD5, RAC2, FTH1, CTD-3252C9.4, FOSB, S100A6, CD37, HPGD, CD83, NFKBID, CAPG, VIM, GCSAML, FOS, ZEB2, TMEM233, CD84, MAOB, IL18, LAPTM5, ARHGEF6, H3F3B, C1orf162, EMP3, RPS26, NFKBIA, ACTG1, ARHGDIB, BIRC3, DUSP6, PTGS2, MYADM, NPDC1, LMNA, HINT1, MARCKSL1, KLK4, NR4A2, SH3BGRL3, RPLP0, HERPUD1, NFIB, PPP1R15A, BTG2, BRI3, SMIM22, TRPM4, RASGEF1B, MARCKS, NUPR1, LAT2, DUSP1, SEMA4A, CEBPD, SNHG19, SEPP1, ABHD2, CLDN3, TM4SF1, CKLF, RHOB, ANXA2, IDS, CALD1, HES4, FABP5, LRRC26, HSD17B6, FASN, KRT18, HEBP2, CTSD, CD24, CLDN4, PLPP1, ELF3, MDK, AZGP1, PRSS23, NUDT8, PLIN2, YWHAZ, SOD2, NKX3-1, RGS2, GLUL, MT2A, LAPTM4A, IFITM3, CNN3, CD82, ID3, MT1E, MT1X, FAM46A, HLA-C, CXCL8, ARHGAP18, ID2, ACPP, CD74, HLA-DRA, HLA-DPB1, TUBA1A, KLK2, SELK, THYN1, IGF1, MT1G, SDCBP, STMN1, RAB27B, TNFAIP3, HSPA6, ADIRF, GNPTAB, C4orf48, HLA-B, PLA2G2A |
| 16 | B_cell | IGKC, IGLC2, IGHA1, CD79A, MS4A1, IGHG1, CD37, CXCR4, JCHAIN, HLA-DRA, LTB, CD74, IGHM, CD83, VPREB3, IRF8, CD52, CD69, HLA-DPB1, GPR183, HLA-DQA1, LAPTM5, CD79B, RP5-887A10.1, HLA-DRB1, LY9, HLA-DQB1, HLA-DPA1, TNFRSF13C, CCR7, CD53, 1-Mar, HLA-DQA2, CD48, POU2F2, CORO1A, CYTIP, ARHGAP24, FCMR, ADAM28, LINC00926, LY86, FCRLA, NCF1, PRKCB, RP11-693J15.5, CLECL1, TNFRSF13B, BLK, SP140, CD22, RCSD1, CYBA, CD55, TMEM154, ACAP1, BIRC3, RAC2, EVI2B, PTPRC, INPP5D, PLAC8, CTSS, MEF2C, BTG1, KLK3, RPS27, RHOH, LSP1, GPSM3, H2AFJ, CST3, PTMS, KLK2, NEAT1, CD9, DSTN, STK17A, PDLIM5, TSC22D3, HLA-DMB, TMEM243, SMAP2, NUPR1, ANKRD44, HLA-B, BANK1, CD63, AREG, TXNIP, HLA-DMA, TRGC1, TSPAN1, NGFRAP1, UQCRQ, MPC2, KRT18, LIMD2, TRPM4, NPDC1, RAC1, HLA-A, FXYD3, APLP2, B2M, KRT8, SOX4, RALGPS2, P4HB, RDH11, TXN, MGST1, TMPRSS2, S100A11, TFF3, IGFBP2, MESP1, HLA-E, PHACTR1, EEF1B2, STEAP2, NR4A2, REL, NKX3-1, NFIB, DHRS7, ARHGDIB, DBI, SPON2, HLA-C, ELF1, ZFP36L2, SAMSN1, BCAM, SEPP1, SPDEF, PMEPA1, BRI3, LRRC26, GAPDH, TMED2, GOLM1, CD151, SORD, SMS, SLC45A3, TSC22D1, HEBP2, TACSTD2, AAK1, KLK4, ISG20, MSMB, AZGP1, KIAA1551, NFKBID, TMEM141, PLPP1, MLPH, ALDH1A3, STK4, TCEB2, BNIP3, TM9SF3, FOXA1, C12orf57, RAB13, SMCHD1, KDELR2, PART1, GDF15, XBP1, PCA3, SEC61G, NEDD4L, DCXR, GLUL, PEBP1, SNHG19, RHOC, CANX, PRDX2, MGST2, ZG16B, SLC39A6, TRPM8, STRA13, CLDN3, ABCC4, FOLH1, FASN, IFI16, CXADR, ST6GAL1, ANAPC11, PERP, TPM1, COX8A, EGR1, NUDT8, EPCAM, PHPT1, SCD, CYB5A, H1F0, HOXB13, PCBD1, GNB2, ADI1, SLC4A4, RRBP1, ACPP, BAIAP2, AR, TSPO, NENF, NME4, MAOA, FIS1, HES4, TCEA3, TNFSF10, PMAIP1, RPS27L, CKB, DHCR24, NDUFB10, MINOS1, KLF4, TRIB1, UCP2, ERGIC1, ACSL3, SPINT2, ELF3, VMP1, ATP5J2, MZT2B, SPOCK1, PPIB, STEAP1, CEBPD, GATA2, ABHD2, ATF3, AGR2, CCND1, SLC9A3R2, CRIP1, MDK, NDUFB4, RAMP1, FAM129A, CCNG2, CLDN4, OR51E2, SP100, C19orf48, SMIM22, MCCC2, CRNDE, KIF22, CTSD, CALD1, PPDPF, SNHG25, PRDM2, APP, ALOX15B, PSAP, CALR, DDT, CPE, HES1, MYO6, LINC01088, TMSB15A, NUCB2, STK17B, PRSS23, ID2, FAM3B, CD59, TNFAIP8, MT1E, CYR61, PPP3CA, TM4SF1, HIST1H4C, MIPEP, EZR, HSD17B6, IFITM3, PTPN1, INSR, BCAS2, TCF4, ZNF331, GPX1, KRT19, PHLDA2, RHOB, IGF1, YPEL5, DRAM2, SNRPN, ITM2B, TSPAN8, ANXA1, MYCBP2, FOS, PRAC1, ATF7IP, RGS2, EIF1AY, HSPH1, FABP5, FNBP1, GPBP1, CXCL8, DNAJA1, PDE4B, VPS37B, MT1G, SKIL, RAB11FIP1, HSPD1 |
| 18 | Chondrocytes | CYTL1, COL2A1, SERPINE2, COL9A3, SCRG1, MSMP, C2orf40, RBP4, C2orf82, TNFRSF11B, FRZB, DCN, CLEC3A, S100A1, LECT1, FGFBP2, SPARC, KCNMA1, TMSB4X, CLU, MT2A, RPL21, TMSB10, S100A6, BGN, PDLIM5, HSPA1B, NEAT1, HMGA1, CALR, JUND, KLK2, ATP5G2, HSPB1, RDH11, FOXP1, HSPA1A, MIF, JUN, FOSB, SOX4, APLP2, YWHAZ, NBEAL1, NUDT4, AC090498.1, KLK3, SMDT1, GPX1, PPP1CB, NPDC1, KLF6, DHRS7, CANX, H2AFJ, DBI, TRGC1, SYNGR2, TSTD1, TSPAN1, MGP, TM9SF3, MARCKSL1, FXYD3, KRT8, MCL1, FOS, NR4A1, SORD, PMEPA1, P4HB, MESP1, HEBP2, CYB5A, IGFBP2, BRI3, TMPRSS2, RHOB, DUSP1, NKX3-1, ERGIC1, ADI1, NME3, KRT18, PSME1, MPC2, EIF1, NFKBIA, PCBD1, HLA-B, STEAP2, RPL4, TSPAN13, ASAH1, TRPM4, SPDEF, SLC45A3, SNHG25, PLPP1, AAK1, SMS, SNRPN, MGST1, MSMB, TSC22D3, TMEM141, ZFP36, DNAJB1, CD46, BCAM, CLDN3, NFIB, NEDD4L, COL9A2, H3F3A, KLK4, SEC11C, PPP3CA, MLPH, FASN, GDF15, TXNIP, AZGP1, TPD52, CLDN4, ZFP36L1, SPON2, FOXA1, CCNG2, TNFSF10, ATF3, ABCC4, ZG16B, BTG2, HLA-C, MARCKS, PART1, DCXR, HSP90AA1, TFF3, SNHG19, LRRC26, FLNB, CXADR, TRIB1, EGR1, ALDH1A3, TRPM8, ZFP36L2, CREB3L4, SCD, SLC4A4, ABHD2, IRF1, FOLH1, DHCR24, PCA3, HES1, HES4, MCCC2, GOLM1, NUDT8, ELF3, ACPP, ZBTB16, HSPH1, TNFRSF12A, REL, BASP1, MDK, PDLIM4, AGR2, SMIM22, OR51E2, INSR, CST3, MIPEP, EMP3, HSD17B6, LINC01088, FAM3B, KLF2, TSPAN8, RGS2, B2M, IGF1, PRAC1, CYR61, IFITM2, HNRNPA1, TNFAIP3, CD74, SRGN, HLA-DPB1, TXN, CHCHD2, HLA-DRA, YBX1, GSTP1, EEF1B2, CXCL8, MT1E, S100A10, ID1, CD63, S100A13, STRAP, ID3, RAN, CD59, GTF2A2, PFDN2, SSBP1, SNRPE, PGAM1, ADRM1, CLTB, ANXA2, EBNA1BP2, SH3BGRL3, PSMB1, EPB41L4A-AS1, KDELR2, HNRNPDL, POMP |

**Table S6.** The results of univariate and multivariate Cox regression analysis of clinical factors and MCMGPS-based risk score in TCGA and GEO-Merged cohorts.

| **Variable** | **TCGA cohort** | | | | **GEO-Merged cohort** | | | |
| --- | --- | --- | --- | --- | --- | --- | --- | --- |
|  | **Univariate** | | **Multivariate** | | **Univariate** | | **Multivariate** | |
|  | HR | *P* | HR | *P* | HR | *P* | HR | *P* |
| **Age**  ≤65 vs >65 | 0.587 | 0.205 | - | - | 1.492 | 0.221 | - | - |
| **Gleason score**  ≤7 vs ＞7 | 4.761 | <0.001 | 2.330 | <0.05 | 3.318 | <0.01 | 2.547 | <0.05 |
| **PSA**  ≤10 vs >10 | 10.686 | <0.001 | 6.915 | <0.01 | 2.059 | <0.05 | 2.045 | <0.05 |
| **Pathologic T**  T1-2 vs T3-4 | 2.596 | <0.05 | 2.032 | <0.05 | 2.723 | <0.05 | 2.314 | <0.05 |
| **Pathologic N**  N0 vs N1 | 1.371 | 0.430 | - | - | - | - | - | - |
| **Risk score**  Low vs High | 5.783 | <0.001 | 4.210 | <0.001 | 22314.360 | <0.001 | 2879.704 | <0.01 |

**Table S7.** The target genes of 10 anti-tumor drugs from the DrugBank database.

| **No**.  **Drugs** | **1** | **2** | **3** | **4** | **5** | **6** |
| --- | --- | --- | --- | --- | --- | --- |
| **Doxorubicin** | TOP2A | NOLC1 | TOP1 | TOP2B |  |  |
| **Bicalutamide** | AR |  |  |  |  |  |
| **Cisplatin** | MPG | A2M | TF | ATOX1 |  |  |
| **Docetaxel** | TUBB1 | MAP2 | MAP4 | MAPT | BCL2 | NR1I2 |
| **Gemcitabine** | RRM1 | TYMS | CMPK1 |  |  |  |
| **Methotrexate** | TYMS | ATIC | DHFR |  |  |  |
| **Paclitaxel** | TUBB1 | BCL2 | MAP4 | MAP2 | MAPT | NR1I2 |
| **Vinblastine** | TUBA1A | TUBB | TUBD1 | TUBG1 | TUBE1 | JUN |
| **Mitomycin C** |  |  |  |  |  |  |
| **Etoposide** | TOP2A | TOP2B |  |  |  |  |
